# Covid-19 diagnosis by combining RT-PCR and pseudo-convolutional machines to characterize virus sequences

**DOI:** 10.1038/s41598-021-90766-7

**Published:** 2021-06-02

**Authors:** Juliana Carneiro Gomes, Aras Ismael Masood, Leandro Honorato de S. Silva, Janderson Romário B. da Cruz Ferreira, Agostinho Antônio Freire Júnior, Allana Laís dos Santos Rocha, Letícia Castro Portela de Oliveira, Nathália Regina Cauás da Silva, Bruno José Torres Fernandes, Wellington Pinheiro dos Santos

**Affiliations:** 1 Escola Politécnica da Universidade de Pernambuco, POLI-UPE, Recife, Brazil; 2 Information Technology Department, Technical College of Informatics, Sulaimani Polytechnic University, Sulaymaniyah, Iraq; 3 Instituto Federal de Educação, Ciência e Tecnologia da Paraíba, Campus Cajazeiras, IFPB, Cajazeiras, Brazil; 4 Departamento de Engenharia Biomédica, Universidade Federal de Pernambuco, DEBM-UFPE, Recife, Brazil

**Keywords:** Machine learning, Molecular medicine

## Abstract

The Covid-19 pandemic, a disease transmitted by the SARS-CoV-2 virus, has already caused the infection of more than 120 million people, of which 70 million have been recovered, while 3 million people have died. The high speed of infection has led to the rapid depletion of public health resources in most countries. RT-PCR is Covid-19’s reference diagnostic method. In this work we propose a new technique for representing DNA sequences: they are divided into smaller sequences with overlap in a pseudo-convolutional approach and represented by co-occurrence matrices. This technique eliminates multiple sequence alignment. Through the proposed method, it is possible to identify virus sequences from a large database: 347,363 virus DNA sequences from 24 virus families and SARS-CoV-2. When comparing SARS-CoV-2 with virus families with similar symptoms, we obtained $0.97 \pm 0.03$ for sensitivity and $0.9919 \pm 0.0005$ for specificity with MLP classifier and 30% overlap. When SARS-CoV-2 is compared to other coronaviruses and healthy human DNA sequences, we obtained $0.99 \pm 0.01$ for sensitivity and $0.9986 \pm 0.0002$ for specificity with MLP and 50% overlap. Therefore, the molecular diagnosis of Covid-19 can be optimized by combining RT-PCR and our pseudo-convolutional method to identify DNA sequences for SARS-CoV-2 with greater specificity and sensitivity.

## Introduction

### Context and motivation

In December 2019, in the city of Wuhan, China, a new virus emerged from the interaction of humans with pangolins and bats. This virus caused a disease that, in mild cases, looked like a simple flu. In severe cases, patients had Severe Acute Respiratory Syndrome, transmitted by the SARS-CoV coronavirus. However, the transmissibility was much higher. In a world connected by modern means of transport and unprepared for a highly transmissible disease, 2019 Coronavirus Disease, Covid-19, transmitted by the new coronavirus, SARS-CoV-2, has spread across the world, generating a world pandemic^1^. The virus can be transmitted by both symptomatic and asymptomatic people. The contagion is given by drops or secretions from sneezing or coughing^2^.

Hospital facilities have been experiencing overcrowding. Most medical professionals are working long hours, and the number of pulmonary ventilators is not enough for all patients. This scenario has led dozens of countries to adopt measures of social isolation and quarantine. They attempt to contain the dissemination, and to mitigate the number of people who need hospitalization^3–5^. The world has been radically changed by measures of confinement and social distancing, while public health systems are being driven to exhaustion. By the end of April 2021, the world registered almost 150 million people infected and more than 3 million dead^6^. Covid-19 manifests itself as a respiratory syndrome. In moderate cases, it manifests clinically as pneumonia. In severe cases, the disease can cause severe respiratory failure, septic shock and/or multiple organ dysfunction (MOD) or failure (MOF)^2,7,8^.

In response to this growing pandemic, several companies and research centers around the world have developed methods to diagnose Covid-19^9^. Rapid tests provide results in about 30 min. Using samples from the patient’s respiratory tract, the Rapid Diagnostic Test (RDT) seeks to detect the presence of antigens. Antigens are substances foreign to the body, causing immune responses. These responses produce specific antibodies, able to bind and interact with the respective antigens, ensuring the protection of the organism. The antibodies are fixed on paper tapes and placed in plastic capsules, similarly to the well-known pregnancy tests. If the target antigen is present in the patient’s sample, it will bind to the antibodies on the tape, generating a visual signal. This method has some limitations. First, detection is only possible in the acute stages of infection, when antigens are expressed. In addition, efficiency depends on factors such as the quality of the sample, the collection protocol, and the formulation of the reagents. It is also common to have false positives, when the antibodies present on the tape recognize antigens from other types of viruses. For these reasons, the sensitivity of the RDT can vary from 34 to $80\%$^10,11^.

Other rapid tests are based on the detection of antibodies in blood samples. However, several studies have shown that the immune response is very weak, late or even absent in many cases of patients confirmed with Covid-19^12–18^. This means that this type of detection is often only possible in recovered patients. Long et al.^19^ reports 285 patients with a positive IgG test. However, these immune responses were observed 19 days after the first symptoms. This condition makes testing ineffective in many situations, as opportunities for treatment and clinical interventions no longer exist. WHO currently does not recommend these types of rapid diagnostic tests for Covid-19. These tests are used in research contexts or as a way of screening patients, or of potential diagnosis^11^.

The standard for the diagnosis of Covid-19 is the molecular diagnosis with Reverse Transcriptase by Polymerase Chain Reaction (RT-PCR)^13,20^. Throat swab samples are usually collected from suspected patients in this type of analysis. The samples are then placed in tubes with virus preservation solutions, where the genetic material of the virus can be extracted. In the first phase, reverse transcription occurs, where a complementary DNA molecule (cDNA) to the virus’s RNA is synthesized. This process occurs through the enzyme DNA polymerase. The RNA is then removed and the Taq DNA polymerase enzyme produces double-stranded DNA, which is a copy of the virus’s RNA. Then, the PCR exponentially amplifies fragments of this DNA during successive cycles, generating millions of copies to be analyzed. Next, the cDNA is aligned with the sequences of the SARS-CoV-2 virus, to analyze the similarity between the sequences. The most used methods are BLAST and FASTA. If there is a match between the two sequences, the patient is confirmed as positive for Covid-19^21–24^.

One of the main limitations of sequence alignment methods is computational complexity and time consumption. In many cases, patients can take days to receive the diagnosis due to sample preparation and genomic analysis. Due to this aspect, several studies have proposed free alignment methods for the classification of genomic sequences. Most of these methodologies involve a method of extracting characteristics, such as the spectral representation of DNA sequences. Thus, the representative attributes of the sequence can be combined with artificial intelligence methods, mainly machine learning. This makes it possible to separate each analyzed sequence into a class (Covid-19 positive or Covid-19 negative, for example)^21,22^.

In this work, we propose a new technique for representing sequences based on the analysis of the relationships between nitrogenous bases. This technique analyzes the DNA sequences obtained by the RT-PCR method, eliminating the alignment process. The DNA sequence is divided into *n* smaller sequences. Each *i*th subsequence is superimposed on a part of the $(i - 1)$th subsequence and on a part of the $(i + 1)$th subsequence, giving rise to two new subsequences. These smaller sequences are represented by $4 \times 4$ co-occurrence matrices with rows and columns corresponding to each of the nitrogenous bases of DNA (Adenine, Cytosine, Timine and Guanine). The co-occurrence matrix considers the occurrence of each of the bases, as well as the relationship between the bases and their immediate neighbors. Then, the co-occurrence matrices are stacked, forming a volume. Considering that the sequences can be subdivided into smaller subsets, with the formation of new co-occurrence matrices, the proposed method has a pseudo-convolutional aspect from an algorithmic point of view. After obtaining the set of matrices, they are concatenated, forming attribute vectors. These extracted attributes correspond to a high-level vector representation of the initial DNA sequence, regardless of the size of the sequence. This feature vector is then classified by machine learning techniques.

Through the proposed method, it is possible to identify virus sequences from a relatively large database. Our proposal is characterized by the following aspects: First, it is not necessary to pre-align the sequence under investigation in relation to the reference sequences; Second, the sequence under study is compared with a wide set of sequences from certain classes, and not just with a reference sequence, reinforcing the test’s reliability. We also emphasize that the method can be applied to sequences of any size, since the representation proposal does not depend on sequence size.

### Related works

Several studies have proposed rapid tests for the diagnosis of Covid-19. The most common methods are based on antibodies^15^. proposed a simple and rapid test for the combined detection of IgG and IgM antibodies. Both antibodies are indicative of infection. However, immunoglobulins M provide an immediate response to viral infections and can be detected within 3 to 6 days after infection. Immunoglobulin G, on the other hand, is important for long-term immunity or for the body’s immune memory. The test was developed to detect IgM and IgG simultaneously in blood samples, allowing detection in a longer time window. For the development of the rapid test, the authors collected samples from eight different laboratories and hospitals in China, with a total of 397 patient samples positive for Covid-19 and 128 negative samples. These results were confirmed by the RT-PCR technique in a sample of the respiratory tract. Blood samples from the patients were pipetted into the test kit, followed by two or three drops of dilution buffer. After 15 min, it was possible to analyze the result using three markers. The first marker (letter C) or line on the display appears in red when the sample is negative. The presence of IgG and IgM is indicated by red or pink lines in the regions with the letters M and G in the kit. Both antibodies may be present in the sample. The tone of the line is also indicative of the concentration level of each type of antibody. The proposed test showed sensitivity of $88.66\%$ and specificity of $90.63\%$. These values can be considered high, compared to results obtained in other studies^25^. The work also tested the performance of the method in 10 patients with peripheral blood. The results remained reliable. Thus, the work is promising and points out an interesting path for a simple and quick diagnosis, which can be an alternative for extensive tests in the population. However, the study does not point to tests with other types of viruses with symptoms similar to the ones provoked by SARS-CoV-2, such as common flu. Given the similarity between viral responses, tests can indicate false positives, in which antibodies bind to SARS-CoV-2-like antigens.

Other works incorporate computational intelligence techniques in Covid-19 diagnosis. Many of them have invested in the automatic classification of X-ray images of the lungs using Deep Learning techniques, mainly Convolutional Neural Networks (CNNs)^26–33^. Apostolopoulos et al.^34^ applied these techniques to distinguish Covid-19 from other lung diseases, such as viral and bacterial pneumonias, pulmonary edema, pleural effusion, chronic obstructive disease, and pulmonary fibrosis. This study used a large database of 3905 X-ray images, including approximately 450 cases of Covid-19. For model training, the images were scaled to 200 $\times$ 200 pixels. The authors also considered image small variations. The images were slightly rotated to make the model robust to variations in position and orientation that may occur in the image acquisition process. In order to extract characteristics from the images, CNN models like Mobile Networks were tested. Three techniques were compared: development of a new CNN architecture; application of a pre-trained CNN (Transfer Learning); and a hybrid method, applying adjustment strategies to specific layers of a pre-trained CNN. The experiments were carried out in Python, using Keras and TensorFlow libraries as back-end. Among the tested configurations, the CNN developed from scratch showed the best results, suggesting that biomarkers related to Covid-19 can be found by the technique. The model achieved an average classification accuracy of $87.66\%$, considering all six classes. With respect to Covid-19, the model achieved $99.18\%$ accuracy, $97.36\%$ sensitivity and $99.42\%$ specificity.

Deep learning architectures have been used to build solutions to combat Covid-19 in many other applications than just chest X-ray or computed tomography diagnostics, such as: (a) epidemiological modeling; (b) search for drugs and active ingredients effective against SARS-CoV-2 infection; (c) prediction of the secondary structure of proteins to support the design of medicines and vaccines; and (d) prediction of severity, recovery and mortality of patients with Covid-19^33,35^.

Gomes et al.^36^ also proposed the use of machine learning techniques for classifying radiographic images, distinguishing between Covid-19, viral pneumonia, bacterial pneumonia, and healthy patients. In contrast to the previous work, the authors invested in low-cost computational methods. The authors tested the Haralick and Zernike moments for attribute extraction and used classic classifiers, such as Multilayer Perceptron neural networks (MLP), Support Vector Machines (SVM), decision trees, and Bayesian networks. The work points out that texture and shpate features can play an important role in Covid-19 image diagnosis. In clinical practice, it is common to find opaque and whitish areas in lung regios affected by pneumonias. Finally, SVM achieved the best overall performance. The authors achieved an average accuracy of $89.78\%$, an average recall and sensitivity of 0.8979, and an average precision and specificity of 0.8985 and 0.9963, respectively. An initial desktop version of the system was developed and made available for free non-commercial use on Github.

Despite the importance of rapid diagnostic methods, WHO recommends the RT-PCR method as the gold standard for determining whether the patient has Covid-19, as RT-PCR can detect the presence of SARS-CoV-2 in the sample blood or secretion^11^. This procedure is similar to that adopted when the SARS outbreak occurred, for the detection of SARS-CoV^37–39^. Several studies and protocols to identify SARS-CoV-2 by molecular diagnosis have already been published^40–44^. Chu et al.^43^ developed RT-PCR assays to detect SARS-CoV-2 in human clinical samples. The authors counted on the first publication of the virus sequence in Genbank, in addition to sequences of other types of coronavirus to make the alignment. They designed two monoplex assays, which target the ORF1b and N gene regions. Then, these primer and probe sequences were confirmed with other released SARS-CoV-2 sequences. The RT-PCR reactions were performed by a thermocycler, using a typical reaction mixture, forward and reverse primers, probe and RNA sample. RNA and DNA purification kits were also used for extraction. The time for each RT-PCR run was about 1 hour and 15 min. To determine the specificity of the assays, they used negative control samples with RNA extracted from other viruses (MERS-CoV, camel coronavirus, influenza A and B, adenovirus, enterovirus, rhinovirus etc.) and from healthy patients. In contrast, all viruses belonging to the subgenus Sarbecovirus (coronavirus similar to SARS-CoV and other coronaviruses) were considered positive in these assays. This decision was made due to the small amount of data available on SARS-CoV-2 at the time of the development of the work. The study tested the method on two patients with suspected SARS-CoV-2 infection. The samples were taken from different locations (sputum vs. smeared throat) and at different periods of infection (day 5 vs. day 3). Both patients received a positive result. Finally, the results of the study demonstrated the clinical value of respiratory samples for the molecular diagnosis of Covid-19. The authors also observed high sensitivity of the N gene to detect the disease, being recommended as a screening test, and Orfb1 as confirmatory. However, RT-PCR is time-consuming and laborious and, as a result, its result may take days to become available^45^. This hampers clinical management and favors the contamination of more people by SARS-CoV-2.

In this sense, the objective of this work is to propose an optimization of the gold standard method, making it possible for a sample suspected of the presence of SARS-CoV-2 to be digitally compared to a list of 24 virus families using a pseudo-convolutional representation for virus DNA sequences and state-of-the-art classifiers.

### Proposed method

**Figure 1:**
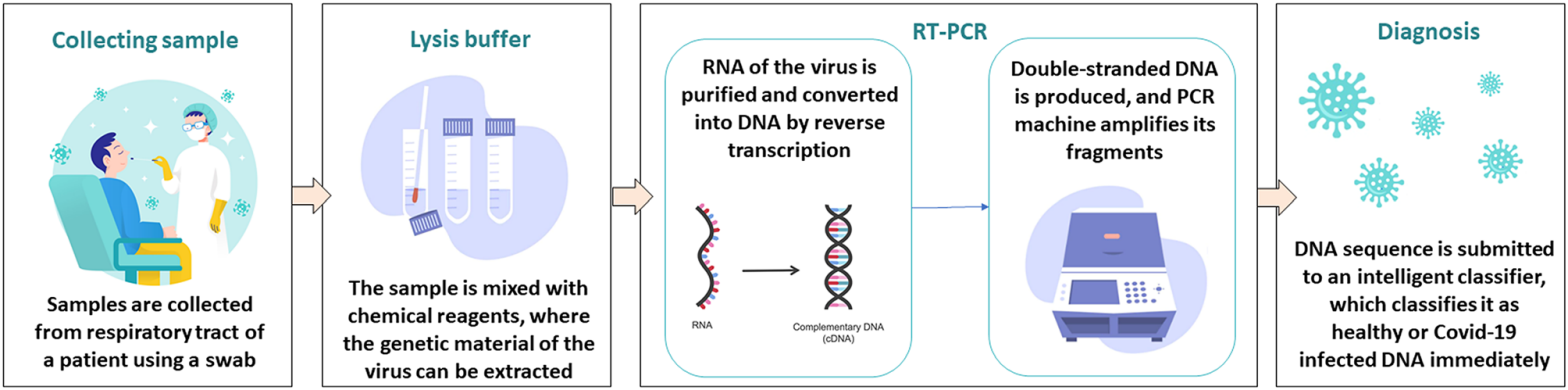
General scheme of the proposal: the RT-PCR process converts and amplify the virus RNA obtained from collected samples and returns a textual DNA sequence as a text file. This textual DNA sequence is converted into a numeric vector by the pseudo-convolutional representation process and then classified into healthy human tissue or one of the 25 virus families or SARS-CoV-2 species by a determined learning machine. The analysis software then returns the diagnostic decision: if the input sample corresponds to a healthy human sample or a determined virus. These virus DNA sequences were obtained from the NIAID Virus Pathogen Database and Analysis Resource—ViPR^46^. Human participants were not involved in our research. No demographic data were collected as well.

**Figure 2:**
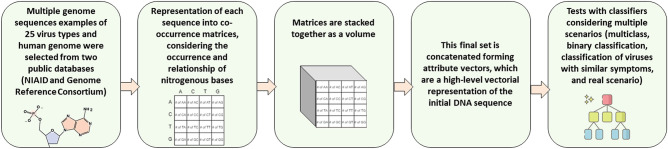
Detailed contribution: 25 classes are used to train the classifiers: 24 virus families and SARS-CoV-2 sequences. Each sequence is analysed, being divided into several sub-sequences considering with a determined degree of overlapping. Each sub-sequence is then represented by $4\times 4$ co-occurrence matrices representing the distribution of nucleotides neighborhoods. The $4\times 4\times m$ volume is then stacked to generate the respective feature vector used as input for the classifier.

In this work, we present a new pseudo-convolutional feature extraction method to represent sequences of nitrogenous bases. Our main objective is to optimize the potential result of RT-PCR for Covid-19 diagnosis. To validate our proposal, we obtained genomic sequences of different viruses from the VIPR (Virus Pathogen Resource)^46^ repository. We employed 24 virus families with more than 500 DNA viral sequences per family, including the Coronaviridae family. SARS-CoV-2 sequences are separated from other Coronaviridae sequences, since we are interested in SARS-CoV-2 identification. Thus, the classification problem involves 25 classes, since SARS-CoV-2 samples are inserted separated from Coronaviridae family. We converted each sequence to numeric vectors using our pseudo-convolutional process. Afterwards, we performed several experiments with different state-of-the-art machine learning methods. Results were evaluated considering four metrics: accuracy, kappa index, sensitivity and specificity. Figure 1 presents a general flow-chart of our proposal, whilst Fig. 2 illustrates our specific contribution.

The pseudo-convolutional representation process is described as follows. Initially, the genome sequence is divided into *n* sub-sequences. These sub-sequences are overlapped with its neighbors. In the overlapping process, a parameter received by the method determines the size of the superimposed pieces. Every *i*th sub-sequence is combined with a piece of the sub-sequence immediately to its left, the $(i - 1)$th, and with the piece at its right, the $(i + 1)$th. An exception is made for the first and the last sequences of the matrix, given that they have only one sub-sequence from which to take a piece. This procedure results into two new sequences for each of the sub-sequences generated from the original genome. After that, these smaller sequences are represented by $4\times 4$ co-occurrence matrices. Each element of the matrix represents the number of occurrences of a given pair of nucleotide bases, as well as the relationship between bases and their immediate neighbors. These elements are AA, AC, AT, AG, CA, CC, CT, CG, TA, TC, TT, TG, GA, GC, GT, and GG. For instance, reading a sequence from left to right, when the present nucleotide base is T and its right neighbor is G, the matrix element in row T and column G is incremented. The final matrix is then normalized, where its maximum value is used as normalization factor. Finally, all the $4\times 4$ matrices are stacked together, forming a $4\times 4\times m$ volume, wherein *m* is the number of sub-sequences resultant from the overlapping process. In general terms:

1 $$\begin{aligned} m = (n-1)\times 2. \end{aligned}$$

**Figure 3:**
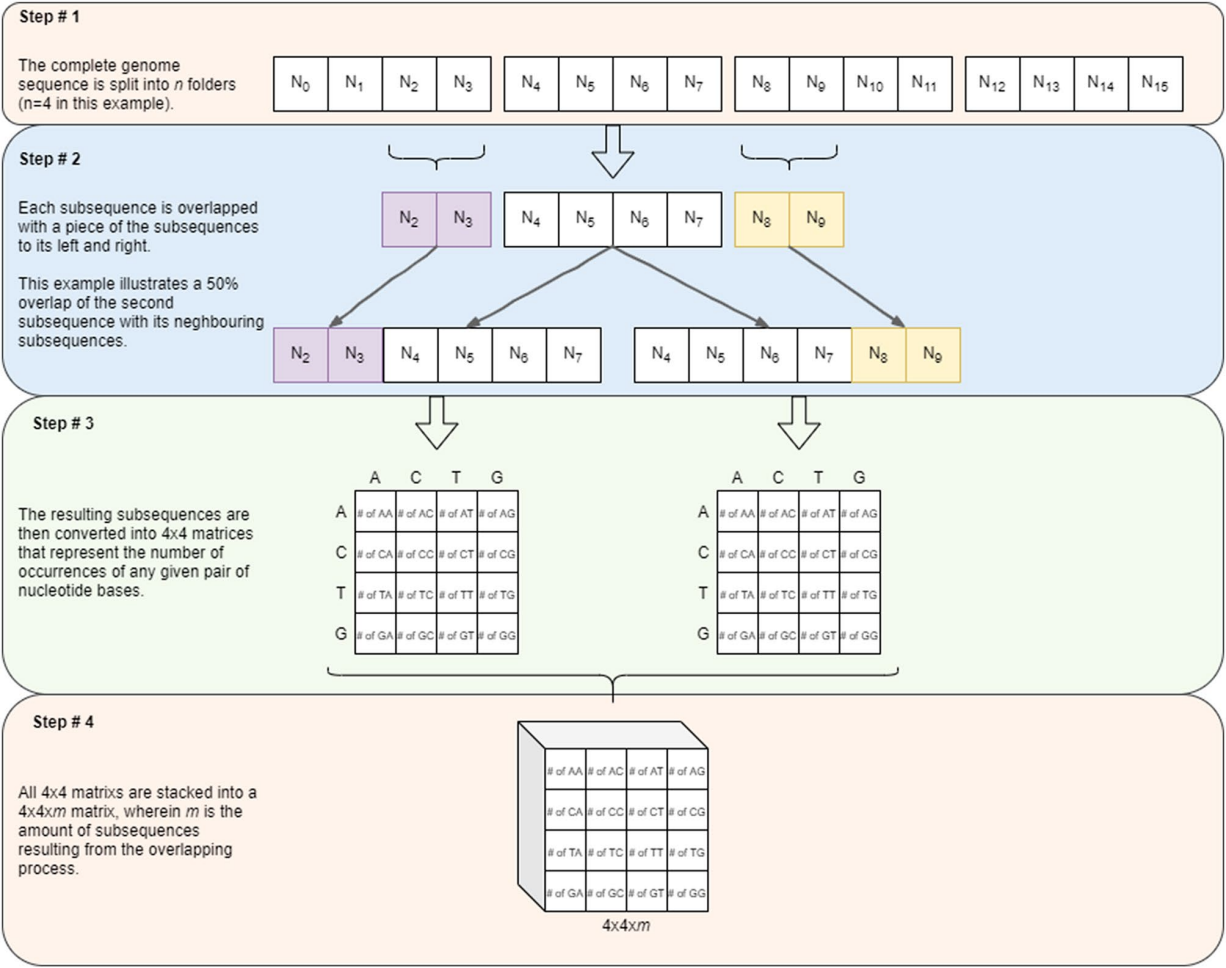
Steps of the proposed method: a new technique for representing genome sequences based on the analysis of the relationship between nitrogenous bases. The complete genome sequence is subdivided into *n* folders. Each sub-sequence is combined with a piece of its neighbors, generating two new sequences. These smaller sequences are represented by co-occurrence matrices, considering the occurrence of each of the nitrogenous bases, and the relationship between bases and their immediate neighbors. In the next step, theses matrices are stacked together as a volume. Finally, this set in concatenated, forming attribute vectors, which are a high-level vectorial representation of the original sequence.

After obtaining this set of matrices, they are then concatenated, forming an attribute vector, i.e. a feature vector. These extracted attributes correspond to a high-level vectorial representation of the initial DNA sequence, independent from its size. The detailed view of this representation process is presented on Fig. 3.

## Results

### Multiclass classification

To assess the efficiency of the proposed method of extracting characteristics, this first round of experiments was conducted in a more challenging scenario with twenty-five different viruses, including SARS-CoV-2. Five types of classifiers were tested: IBk, Multilayer Perceptron (MLP), Naive Bayes classifier (NBC), Random Forest and Support Vector Machines (SVM). All experiments were carried out with the Weka data mining Java library. The parameters used in each machine learning method are shown in Table 1.

**Table 1:** Classifiers parameters: SVMs with linear kernel; MLPs with 48 neurons in the hidden layer; random forests with 100 trees; and standard IBK and Bayesian networks.

Classifier	Hyperparameters
Random Forest (RF)	Number of estimators: 100
Naive Bayes Classifier (NBC)	–
IBK	Number of neighbors to use: 1
	Distance metric: Euclidean distance
Multilayer Perceptron (MLP)	Learning rate: 0.3
	Momentum: 0.2
	Single hidden layer with 48 neurons (number attributes divided by two)
	Sigmoid activation function
Support Vector Machine (SVM)	C: 0.1
	Linear Kernel

**Figure 4:**
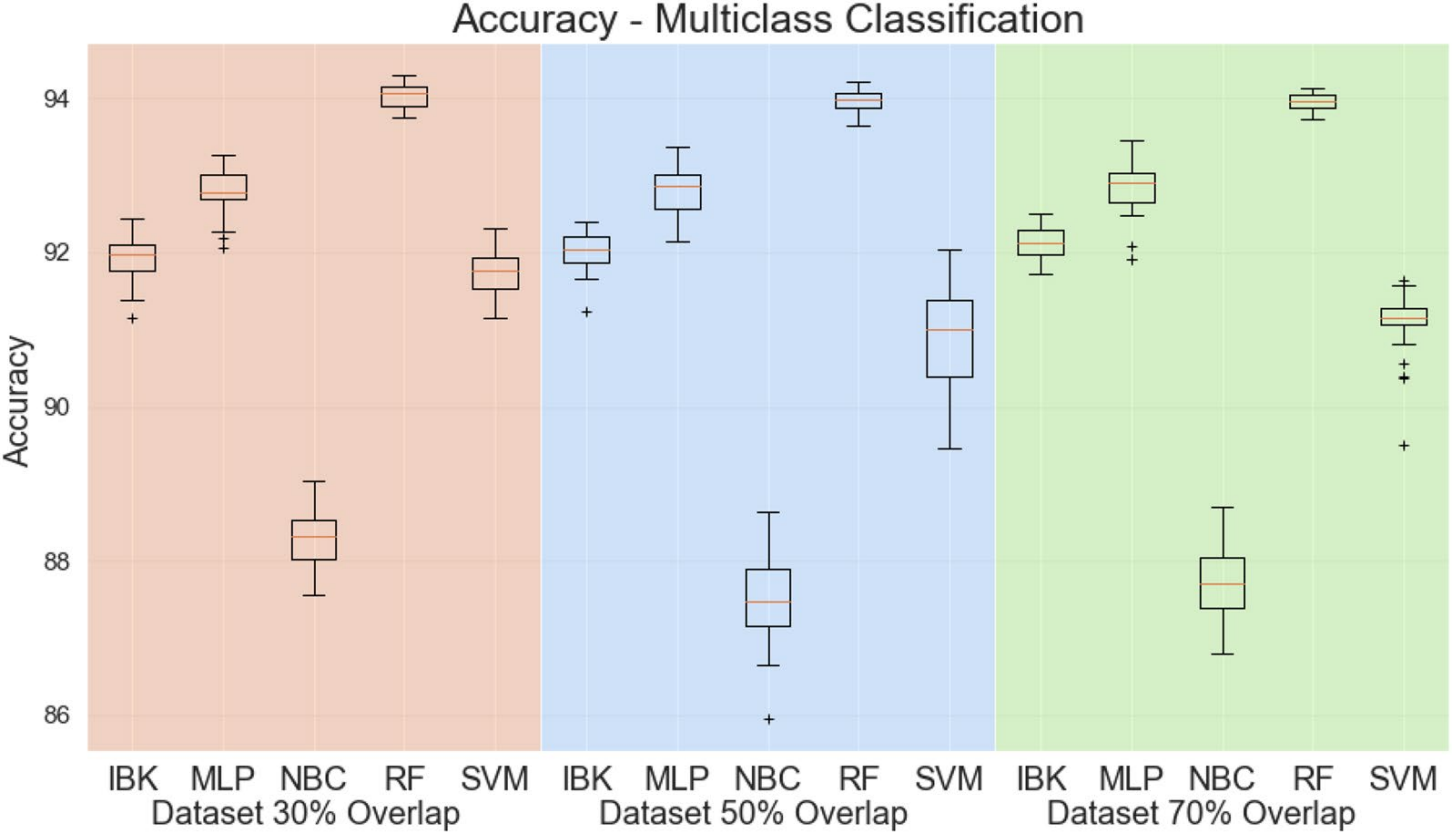
Accuracy for multiclass scenario.

Figure 4 shows the accuracy for all classifiers in the datasets with 30%, 50%, and 70% of overlap, respectively. Considering this multiclass classification, all three datasets (with $30\%$, $50\%$, and $70\%$ overlap) presented Random Forest classifier with the highest accuracies (approximately 94% in all the datasets).

**Figure 5:**
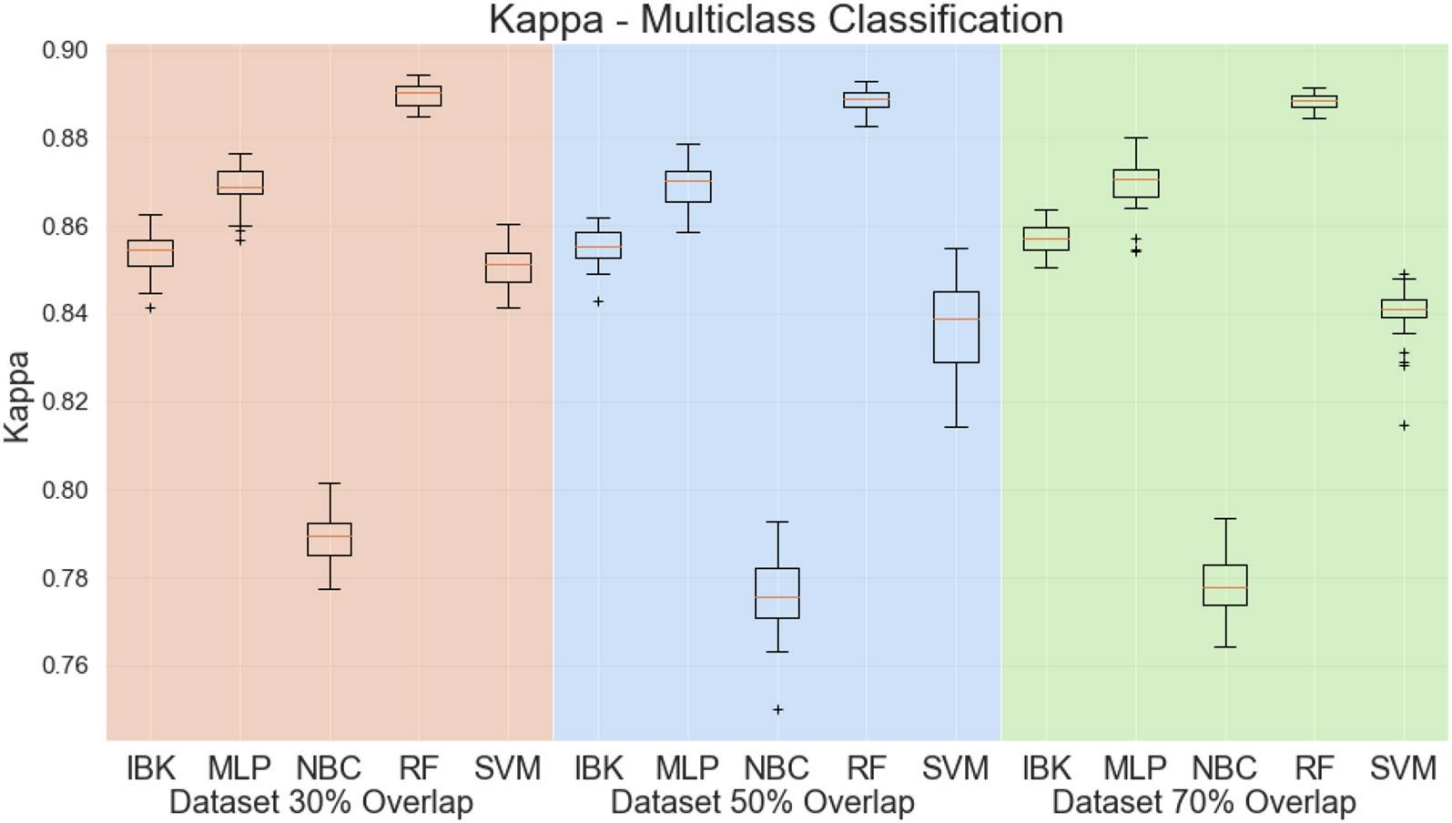
Kappa statistic for multiclass scenario.

Figure 5 shows box plots for the Kappa statistic. Since Kappa statistic is less sensitive to the high imbalanced test dataset, it is a better evaluation metric then accuracy. Nevertheless, the Random Forest classifier achieves the highest Kappa statistics compared with the other classifiers (above 0.88 in all experiments).

**Figure 6:**
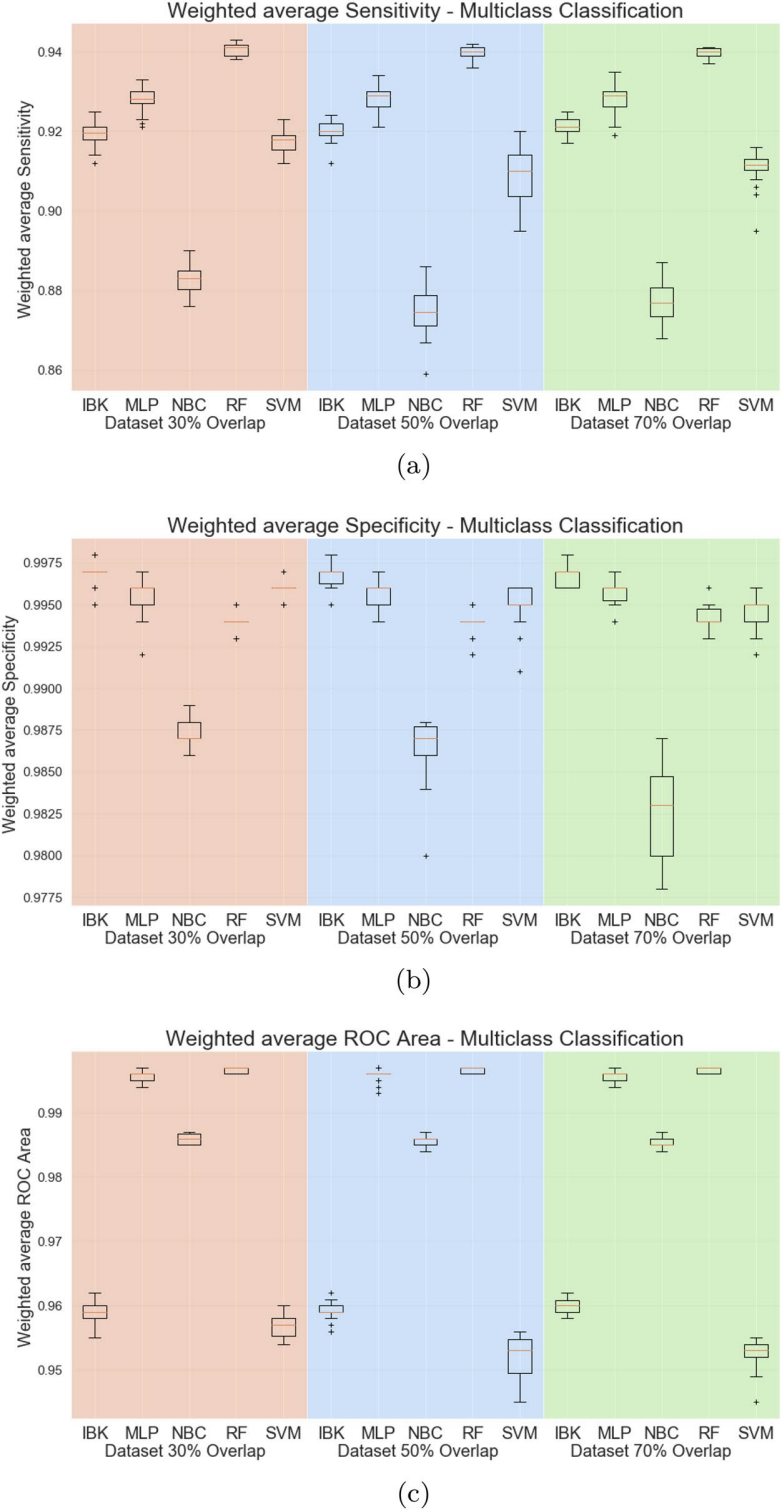
Weighted average sensitivity (**a**), specificity (**b**), and ROC area for Multiclass test scenario.

Figure 6 presents the weighted average sensitivity, specificity, and ROC area for all datasets and classifiers. For the weighted average sensitivity and ROC area, Random Forest results are higher or at least equal to other classifiers. For the weighted average specificity, visual analysis of Fig. 6b suggests that the IBK classifier achieves higher scores on this metric. However, all classifiers, except Naive Bayes Classifier, achieved results above 0.99 on weighted average specificity. Therefore the Random Forest is presented as a robust classifier for this task.

**Figure 7:**
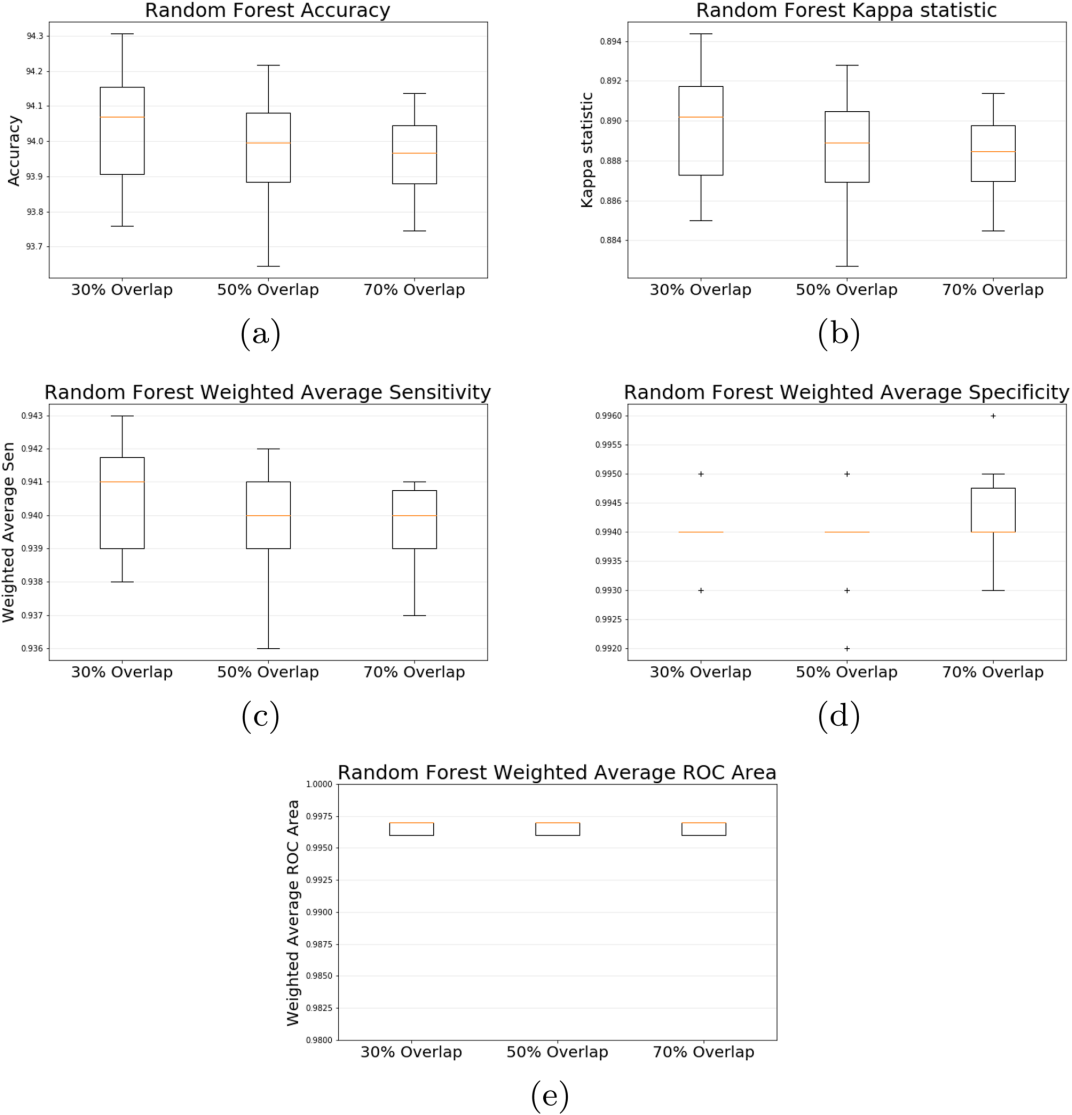
Random Forest accuracy (**a**), kappa (**b**), weighed average sensitivity (**c**), specificity (**d**) and ROC area (**e**) in 30%, 50% and 70% overlap percentages.

Aiming to evaluate the overlap percentage in the feature extraction method, Fig. 7 shows box plots for accuracy, Kappa statistic, weighted average precision, recall and ROC area for the Random Forest classifier in the datasets with 30%, 50%, and 70% overlap percentages. The variance of accuracy and kappa in the dataset with 30% overlap is higher than in the 50% and 70% overlap dataset. However, 30% overlap seems to be slightly better, or at least at the same level, as the others overlap percentages.

Due to class imbalance in the test dataset, we need to evaluate sensitivity, specificity, and ROC area for each class separately. Considering the Random Forest classifier in the dataset with 30% overlap, Table 2 shows the results of sensitivity, specificity, and ROC area individually for each virus in the database. Specificity and ROC Area results are above 0.9 for every virus. The sensitivity varies from 0.99391 for Pneumoviridae to 0.23397 for Filoriviridae. However, for most of the classes, sensitivity has values greater than 0.8, including SARS-CoV-2 class with a sensitivity of 0.82.

**Table 2:** Random Forest sensitivity, specificity, and ROC area for every single class (results from dataset with 30% overlap).

Class	Sensitivity-Recall		Specificity		ROC Area	
	Average	Std. Dev.	Average	Std. Dev.	Average	Std. Dev.
Class 0—Picomaviridae	0.46387	0.01407	0.99674	0.00028	0.99223	0.00163
Class 1—Arenaviridae	0.44609	0.01301	0.99906	0.00008	0.99773	0.00121
Class 2—Caliciviridae	0.98755	0.00392	0.99886	0.00045	0.99893	0.00089
Class 3—Pneunioviridae	0.99391	0.00285	0.99999	0.00001	0.99993	0.00025
Class 4—Phenuiviridae	0.94210	0.00704	0.99966	0.00005	0.99840	0.00076
Class 5—Togaviridae	0.99159	0.00327	0.99958	0.00016	0.99950	0.00067
Class 6—Poxviridae	0.99314	0.00662	0.99994	0.00003	0.99947	0.00191
Class 7—Filoviridae	0.23397	0.03284	0.99950	0.00005	0.99877	0.00096
Class 8—Flaviridae	0.55250	0.00768	0.98242	0.00144	0.95860	0.00308
Class 9—Hantaviridae	0.98284	0.00565	0.99960	0.00004	0.99907	0.00100
Class 10—Lassa virus	0.59120	0.03118	0.99778	0.00006	0.99707	0.00112
Class 11—Dengue	0.87925	0.01729	0.98384	0.00029	0.98910	0.00070
Class 12—Hepeviridae	0.98924	0.00711	0.99972	0.00014	0.99933	0.00083
Class 13—Ebola virus	0.39652	0.05417	0.99913	0.00004	0.99833	0.00094
Class 14—Enterovirus	0.70652	0.02068	0.99144	0.00021	0.99313	0.00034
Class 15—Zika virus	0.96675	0.01076	0.99728	0.00002	0.99800	0.00000
Class 16—Nairoviridae	0.98687	0.00686	0.99974	0.00008	0.99843	0.00238
Class 17—Coronaviridae	0.96194	0.00181	0.99988	0.00005	0.99833	0.00119
Class 18—Pararnyxoviridae	0.98781	0.00274	0.99982	0.00006	0.99970	0.00078
Class 19—Rhabdoviridae	0.97468	0.00541	0.99976	0.00009	0.99967	0.00047
Class 20—Hepatitis C virus	0.97425	0.00244	0.99288	0.00074	0.99823	0.00062
Class 21—Peribunyaviridae	0.95344	0.00603	0.99870	0.00046	0.99523	0.00138
Class 22—Reoviridae	0.98059	0.00261	0.99923	0.00014	0.99963	0.00048
Class 23—Phasrna Viridae	0.29167	0.10704	0.99999	0.00000	0.99000	0.01464
Class 24—SARS-Cov2	0.82222	0.05613	0.99974	0.00001	0.99883	0.00250

**Figure 8:**
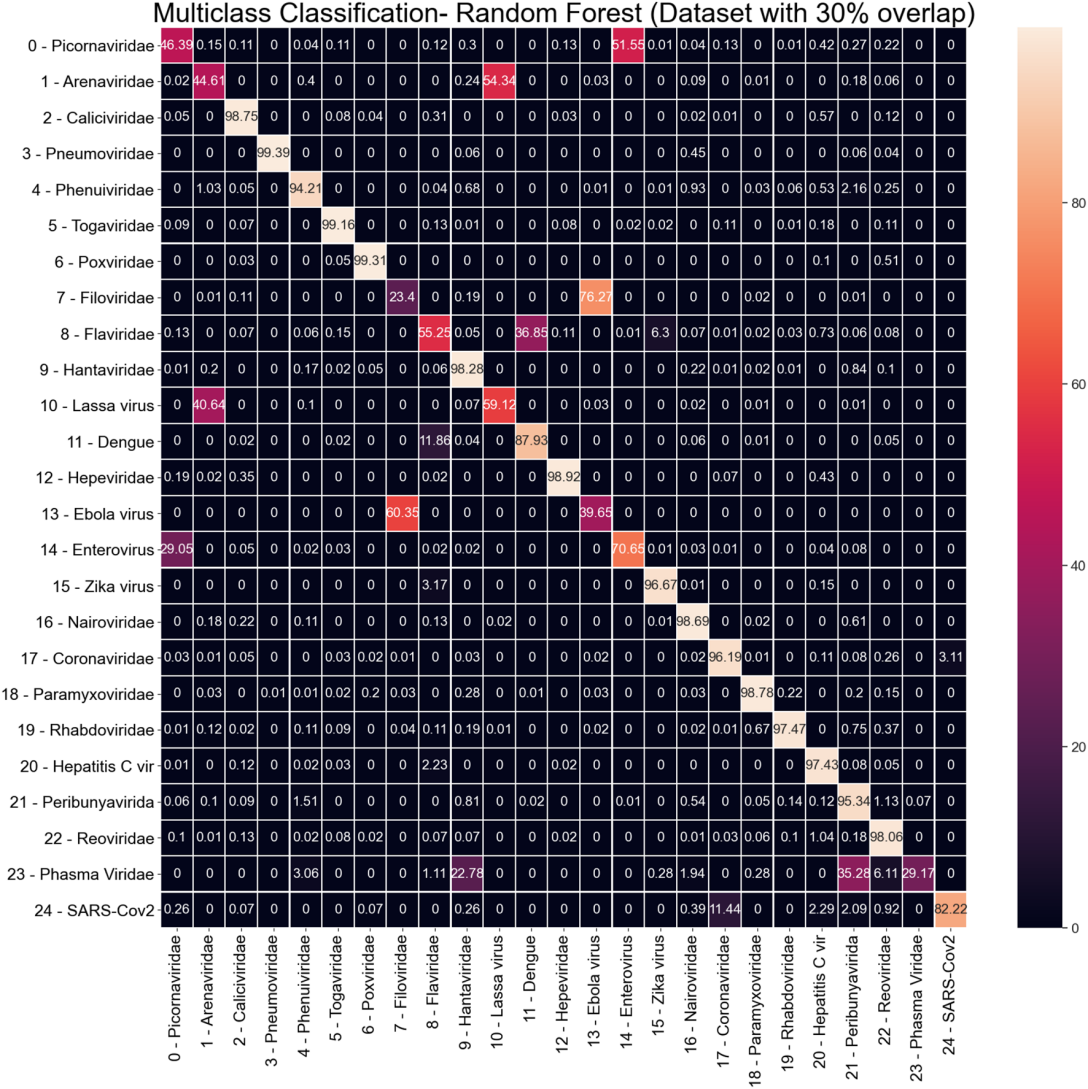
Random Forest average Confusion Matrix (results from dataset with 30% overlap).

In order to perform a visual analysis of these results, Fig. 8 shows the average confusion matrix for the Random Forest classifier in the dataset with 30% overlap. The confusion matrix is expressed in terms of percentage for the particular class, and the classes indexes numbers are the same as shown in Table 2. We can see that, for some classes, there is a confusion with another virus. For example, most of the Picornaviridae virus (index 0) is classified as Enterovirus (index 14). This confusion is not symmetrical: Picornaviridae is misclassified as Enterovirus, but Enterovirus is not misclassified as Picornaviridae. The only exception for this observation of confusion with another virus type is the Phasma Viridae (index 23), which is confused with two other viruses: Hantaviridae (index 9), and Peribunyaviridae (index 21). However, since there are few examples of Phasma Viridae in the dataset (only 42 examples), those results may be caused by the low representative of this class in the dataset.

Regarding the SARS-CoV-2 virus (index 24), the only relevant confusion is with Coronaviridae (index 17). It is a predictable outcome since SARS-CoV-2 belongs to the Coronaviridae virus family. 3.1% of Coronaviridae examples are classified as SARS-CoV-2, i.e. the only confusion noticed in column 24 of the confusion matrix. A more significant confusion is noticed between SARS-CoV-2 and Coronaviridae since 11% of SARS-CoV-2 are misclassified as Coronaviridae.

**Figure 9:**
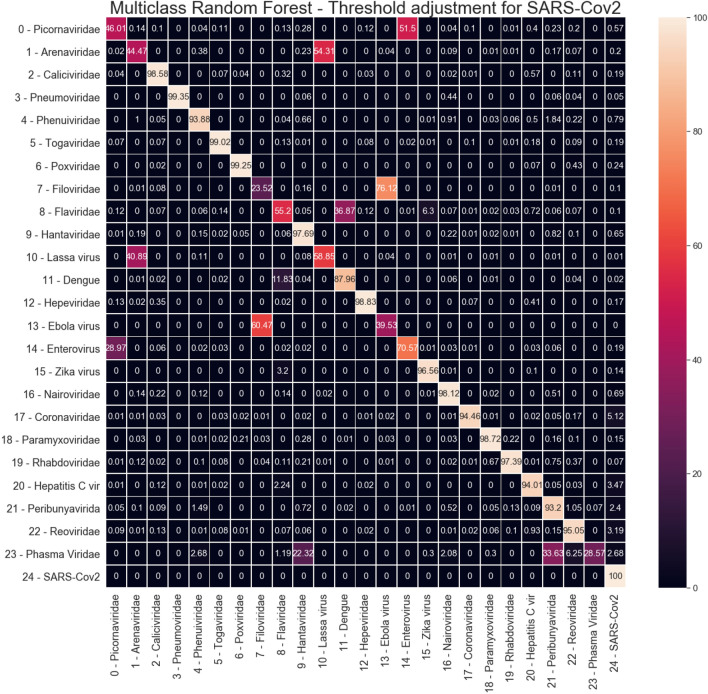
Random Forest average Confusion Matrix with threshold adjustment for 100% sensitivity on SARS-CoV-2 (index 24).

Since the ROC area for SARS-CoV-2 is 0.99883 (Table 2), we performed a threshold adjustment for SARS-CoV-2 class in order to reach 100% sensitivity. The new average confusion matrix is shown in Figure 9. Higher false positives for SARS-CoV-2 remains from Coronaviridae (5.1% - index 17). In the sequence of false positive rates, we have: Hepatitis C virus (3.47% - index 20), Reoviridae (3.19% - index 22), and Phasma Viridae (2.68% index 23).

### Binary classification

Since the highest false positives for SARS-CoV-2 are from Coronaviridae in the multiclass scenario, we evaluated the same classifiers for a binary classification between Coronavirus and SARS-CoV-2. For this experiment, only the dataset with 30% overlap was used, since this overlap percentage has shown to represent the virus genome sequences satisfactorily.

**Figure 10:**
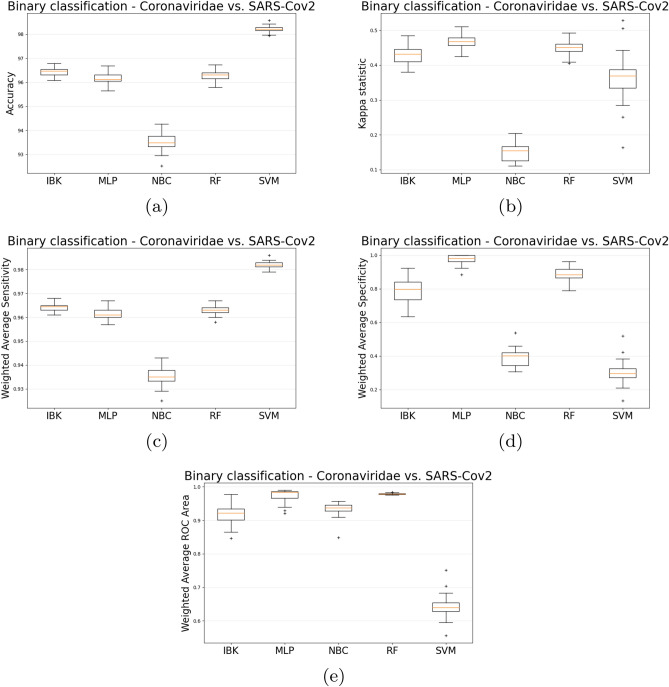
Binary classification (Coronavuris vs. SARS-CoV-2 using the 30% overlap dataset) accuracy (**a**), kappa (**b**), weighed average sensitivity (**c**), specificity (**d**), and ROC area (**e**).

Figure 10 shows the accuracy, kappa statistic, weighted average sensitivity, specificity, and ROC area for each classifier. It is important to state that there is still a relevant imbalance between the number of Coronaviridae and SARS-CoV-2 examples in the dataset (3256 and 171, respectively). So, the Kappa statistic is still more appropriate than accuracy to assess the classifier’s overall performance. Regarding Kappa statistics, weighted average specificity, and ROC area, MLP results are higher or equal to other classifiers. For the weighted average sensitivity, SVM achieves higher results than MLP. Nevertheless, given that average sensitivity for MLP is higher than 0.96 and MLP overcomes SVM in all other metrics, MLP seems to be a more robust classifier for this particular task.

**Figure 11:**
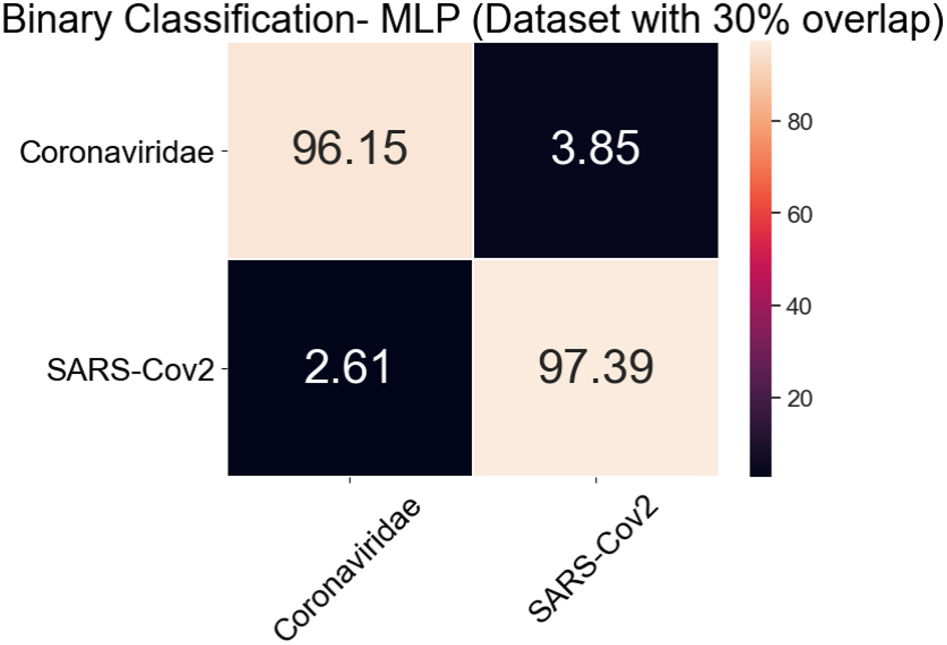
MLP average Confusion Matrix for binary classification task (Coronaviridae vs. SARS-CoV-2 using the 30% overlap dataset).

Table 3 shows the sensitivity, specificity and ROC Area for each class. It is possible to notice that each one of those metrics has values above 0.96. Figure 11 shows the average Confusion Matrix for MLP classifier. There was no relevant difference with the multiclass scenario regarding the confusion between Coronaviridae and SARS-CoV-2 since there is still a 3.85% of Coronaviridae examples misclassified as SARS-CoV-2. However, about the confusion between SARS-CoV-2 and Coronaviridae, the binary MLP classifier achieved 2.61% of confusion while 11% in the multiclass scenario.

**Table 3:** Results of Sensitivity, specificity, and ROC area for MLP binary classifier (Coronavirus vs. SARS-CoV-2 using the 30% overlap dataset).

Class	Sensitivity-Recall		Specificity		ROC Area	
	Average	Std. Dev.	Average	Std. Dev.	Average	Std. Dev.
Class 17 Coronaviridae	0.96151	0.00246	0.97386	0.03052	0.97353	0.01863
Class 24—SARS-Cov2	0.97386	0.03052	0.96151	0.00246	0.97353	0.01863

### Viruses with similar symptoms

In this experiment, viruses were selected due to similar symptoms. The dataset was arranged into four classes: SARS-CoV-2, Coronaviridae, Paramyxoviridae, and Miscellaneous. The Miscellaneous Class is a compound of Peneumoviridae, Hantaviridae, Enterovirus, and Nairoviridae. Then, the same classifiers used previously were evaluated in this classification task.

**Figure 12:**
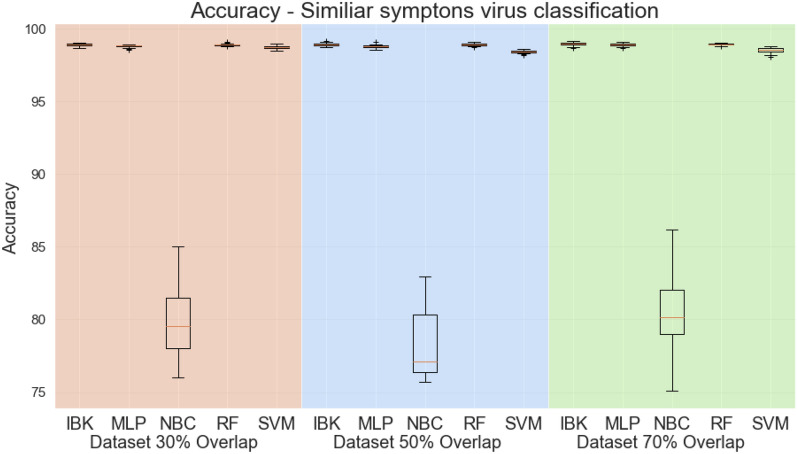
Accuracy for similar symptoms scenario.

**Figure 13:**
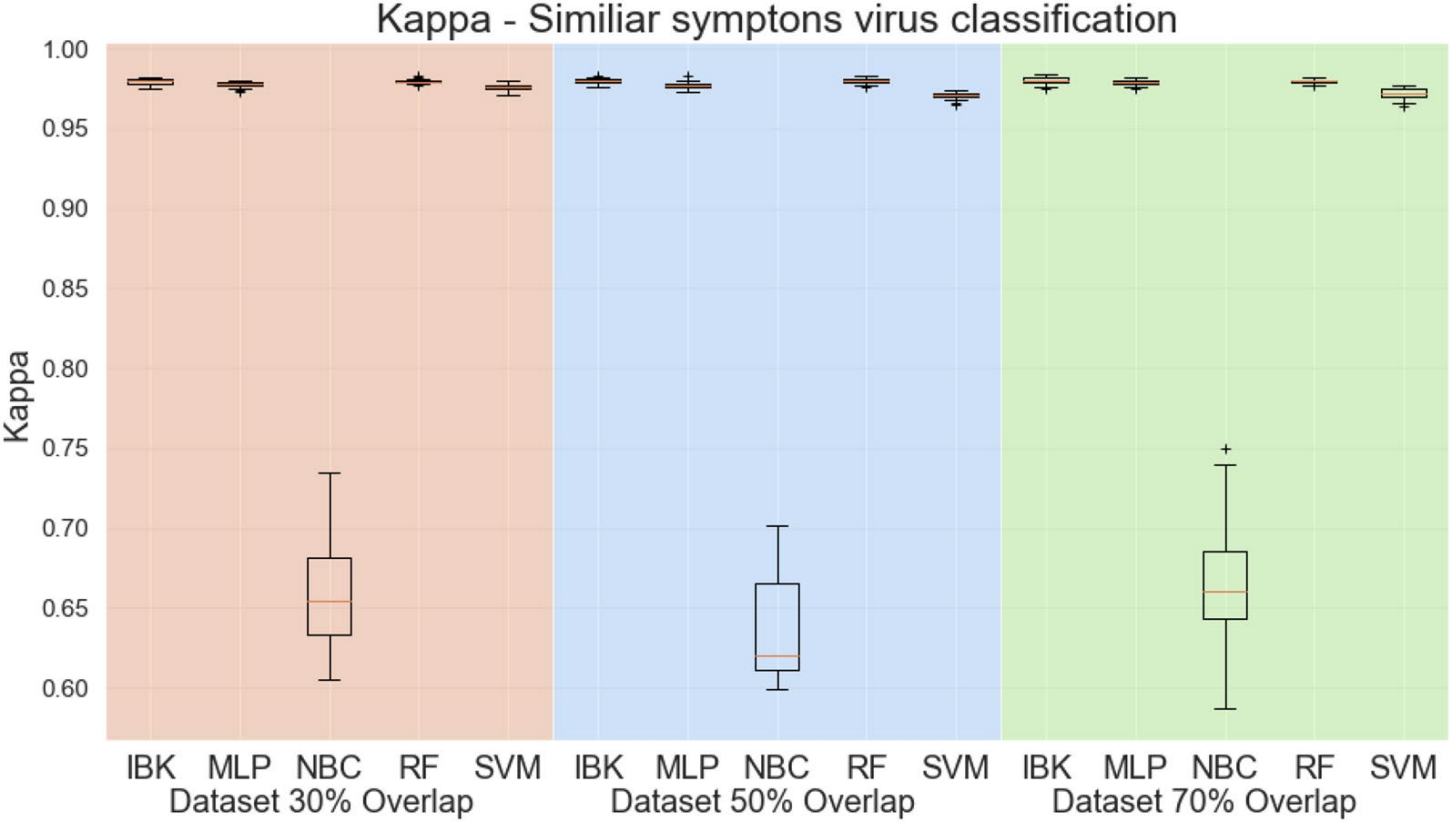
Kappa Statistic for similar symptoms scenario.

**Figure 14:**
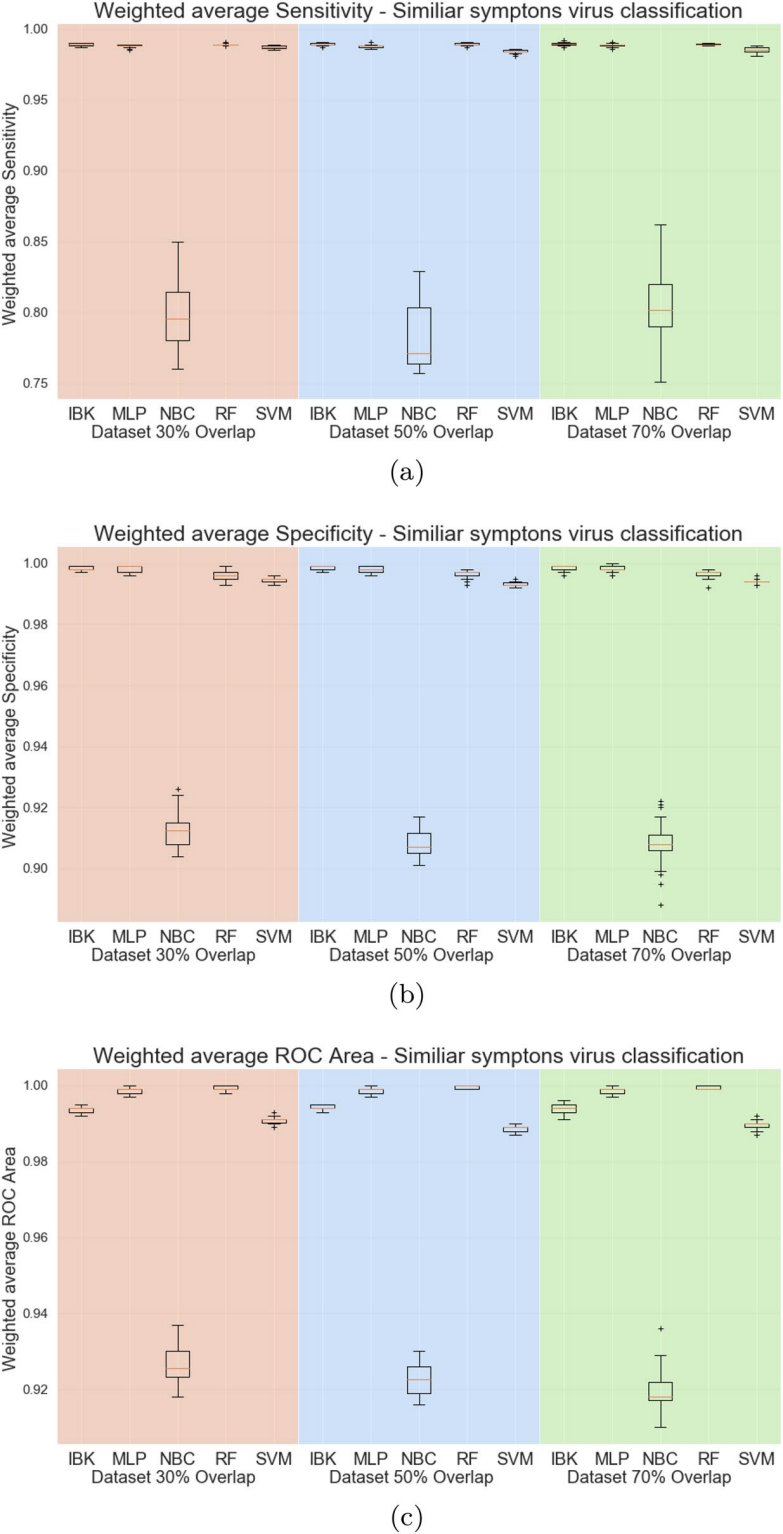
Weighted average sensitivity (**a**), specificity (**b**), and ROC area for similar symptoms viruses test scenario.

Figures 12 and 13 shows the accuracy and kappa for all classifiers and datasets in this classification task. Except for the Naive Bayesian classifier, classifiers have similar performance metrics, with approximately 97% accuracy and kappa equal to 0.96. Figure 14 shows the weighted average specificity and sensitivity and ROC are. The weighted average sensitivity and specificity look very similar to all classifiers (except Naive Bayes Classifier). However, the weighted average ROC areas for MLP and Random Forest classifiers are slightly higher than the other classifiers, although IBK and SVM classifiers also achieved weighted average ROC areas above 0.98 in all datasets.

**Figure 15:**
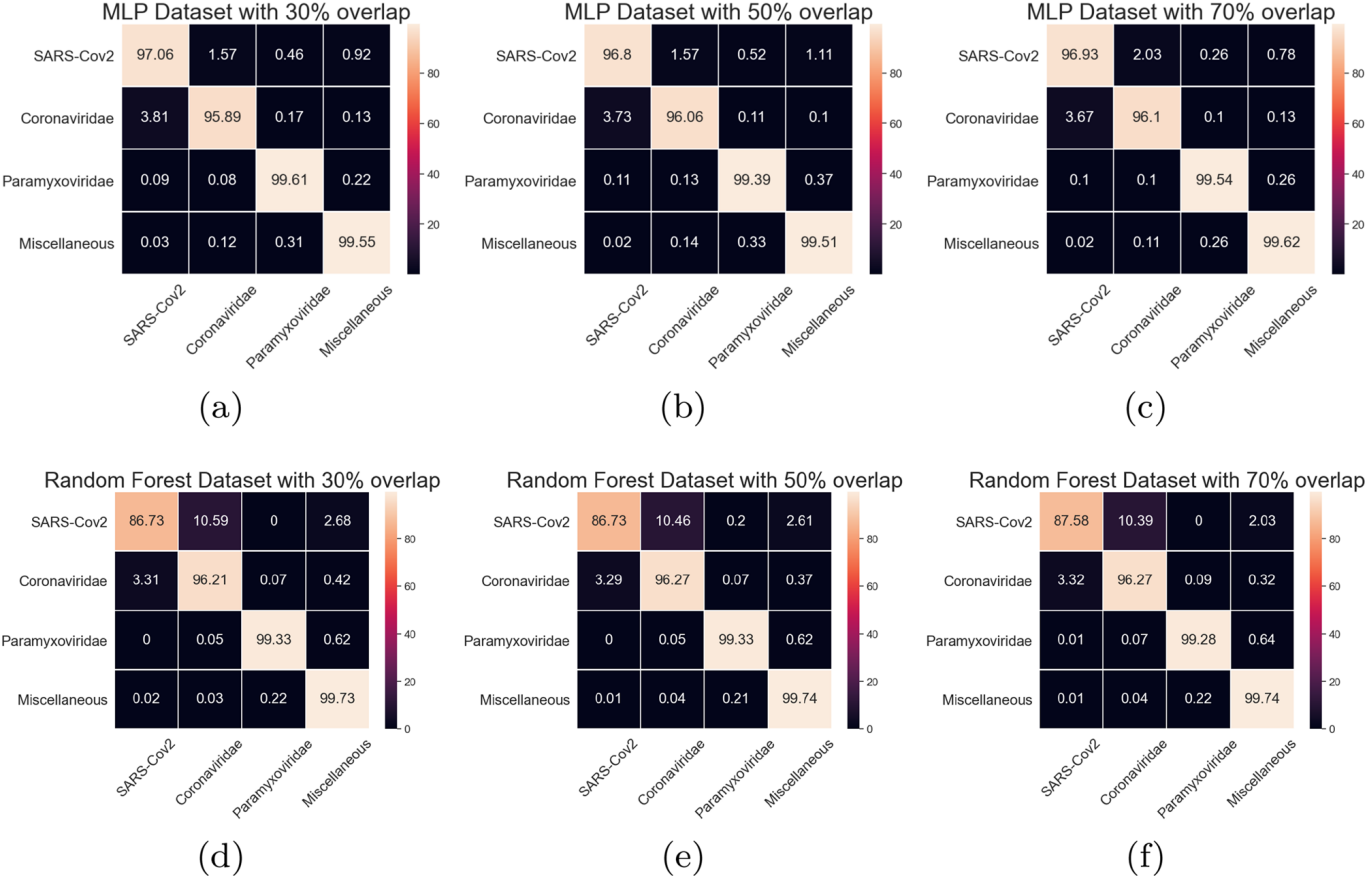
Average Confusion matrices for MLP and Random Forest in the Similar symptoms viruses test scenario.

In order to better evaluate the MLP and Random Classifier, Fig. 15 shows the confusion matrices for those classifiers in all datasets. The Random Forest presents a confusion between the SARS-CoV-2 and the Coronaviridae of approximately 10%. It is very similar to the achieved results in the multiclass scenario. However, the MLP classifier achieved significantly low confusion between SARS-CoV-2 and Coronaviridae (1.57% in the datasets with 30% and 50% overlap). The main confusion found in the MLP classifier is between Conronarividae and SARS-CoV-2 (3.81% for the dataset with 30% overlap). By MLP confusion matrix analysis is not possible to find significant differences between the 30%, 50%, or 70% overlap percentages. Since the 30% overlap requires less computational effort to extract the features, we can select the MLP classifier with a 30% overlap dataset as a better approach to this particular task. Table 4 shows the sensitivity, specificity and ROC area for each class. The average ROC Area and specificity are above 0.99 for all classes. The average sensitivity is also above 0.99 for the Paramyxoviridae and Miscellaneous classes. The lowers sensitivity is for Coronaviridae (0.959), while a slightly higher sensitivity is achieved for SARS-CoV-2 (0.97).

**Table 4:** Results of Sensitivity, specificity, and ROC area for MLP classifier in the similar symptoms viruses test scenario (results from dataset with 30% overlap).

Class	Sensitivity-Recall		Specificity		ROC Area	
	Average	Std. Dev.	Average	Std. Dev.	Average	Std. Dev.
SARS-Cov2	0.97059	0.03387	0.99187	0.00046	0.99583	0.00481
Coronaviridae	0.95891	0.00249	0.99882	0.00069	0.99687	0.00076
Pararnyxoviridae	0.99611	0.00430	0.99726	0.00104	0.99863	0.00244
Miscellaneous${}^{\mathrm{a}}$	0.99548	0.00151	0.99827	0.00153	0.99943	0.00072

${^{\mathrm{a}}}$Miscellaneous class includes four virus types: Pneunioviridae, Hantaviridae, Enterovirus, and Nairoviridae.

### Real test scenario

In this scenario, the SARS-CoV-2 test is designed as a three-class classification problem: SARS-CoV-2 (the test target), GRCh38 (the healthy human reference), and Coronaviridae (a virus control sample). The same classifiers used in the other experiments were applied to this new task.

**Figure 16:**
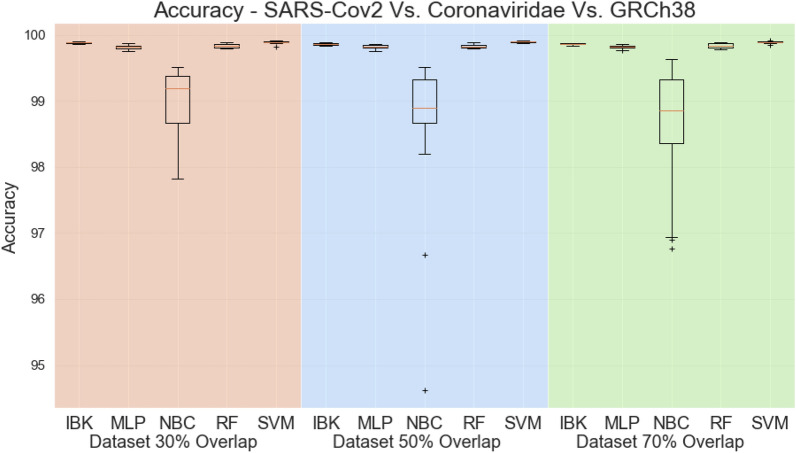
Accuracy for SARS-CoV-2 test scenario.

**Figure 17:**
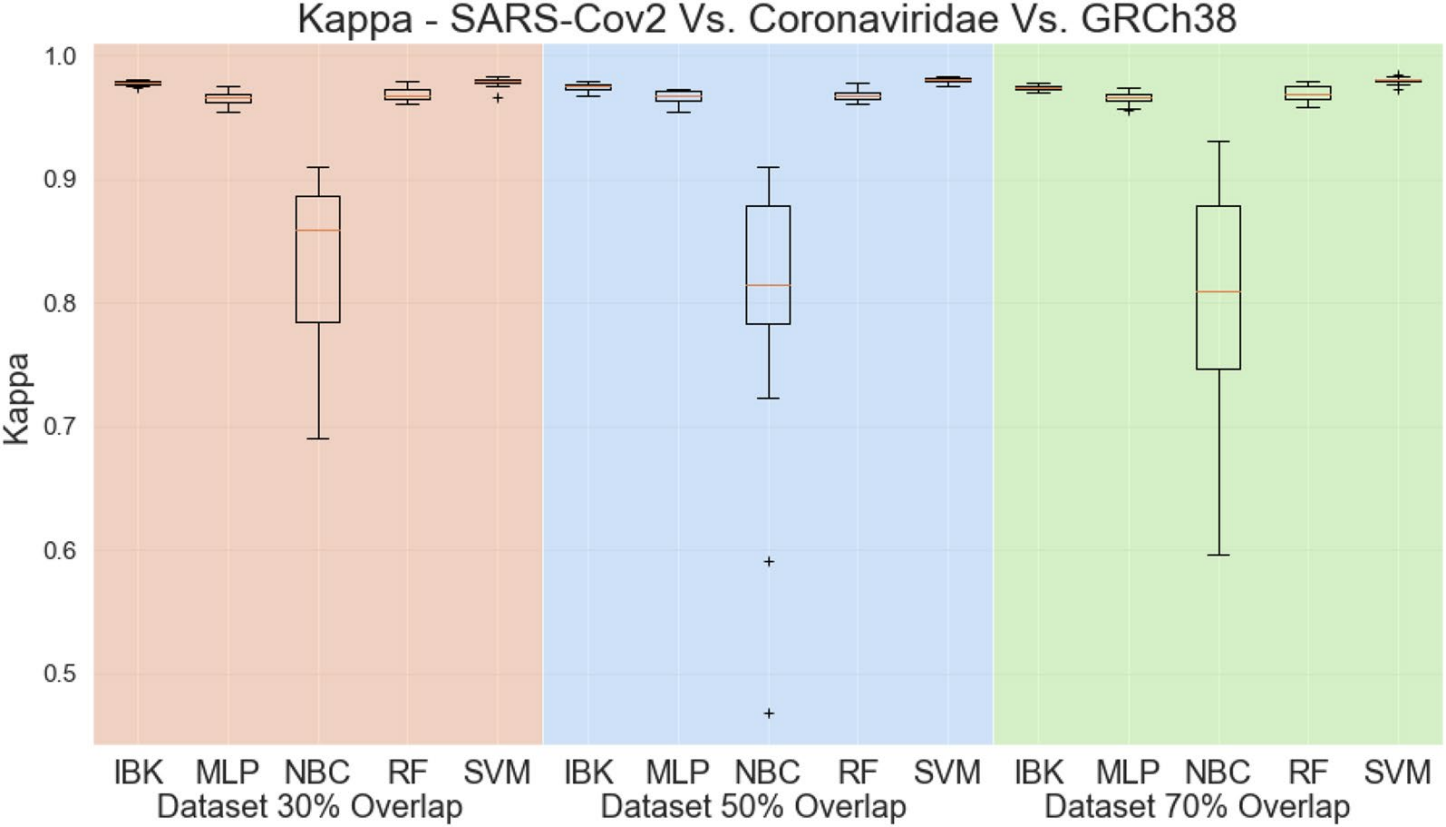
Kappa Statistic for SARS-CoV-2 test scenario.

**Figure 18:**
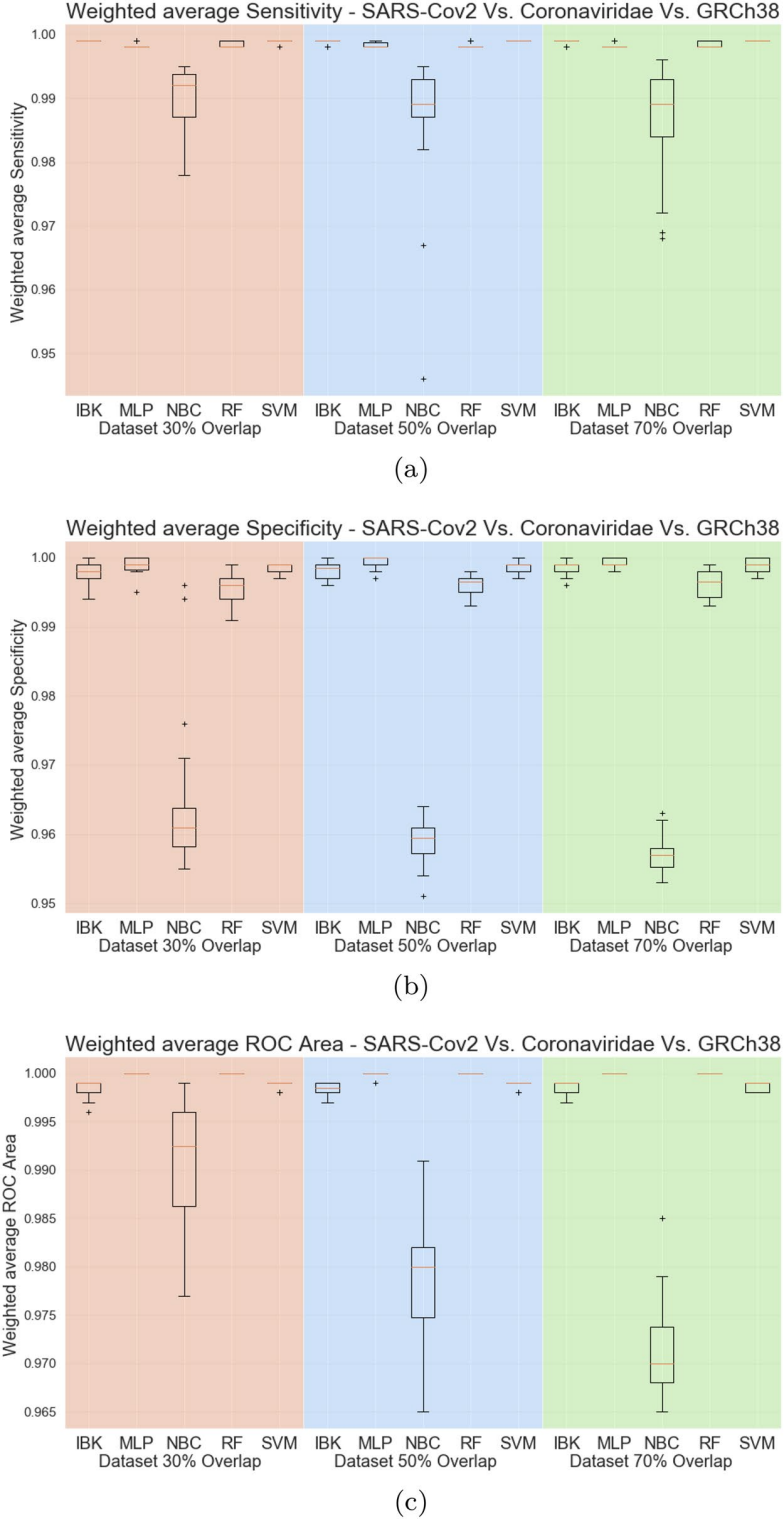
Weighted average sensitivity (**a**), specificity (**b**), and ROC area for SARS-CoV-2 test scenario.

Figure 16 shows the accuracy, whilst Fig. 17 presents the kappa statistic results. Except for the Naive Bayes Classifier, all other classifiers achieved accuracies higher than 99% and kappa indeces above 0.9. By these metrics, it is not possible to distinguish the best classifier. The same behavior is observed in the weighted average metrics shown in Fig. 18. Weighted average sensitivity, specificity, and ROC area are higher than 0.99 for all classifiers except the Naive Bayes Classifier.

**Table 5:** Results of sensitivity, specificity, and ROC area for MLP classifier in the SARS-CoV-2 test scenario (results from dataset with 50% overlap).

Class	Sensitivity-Recall		Specificity		ROC Area	
	Average	Std. Dev.	Average	Std. Dev.	Average	Std. Dev.
SARS-Cov2	0.98824	0.01198	0.99860	0.00020	0.99947	0.00056
Coronaviridae	0.96196	0.00190	0.99967	0.00017	0.99810	0.00094
GRCh38	0.99923	0.00028	0.99928	0.00094	0.99997	0.00018

**Figure 19:**
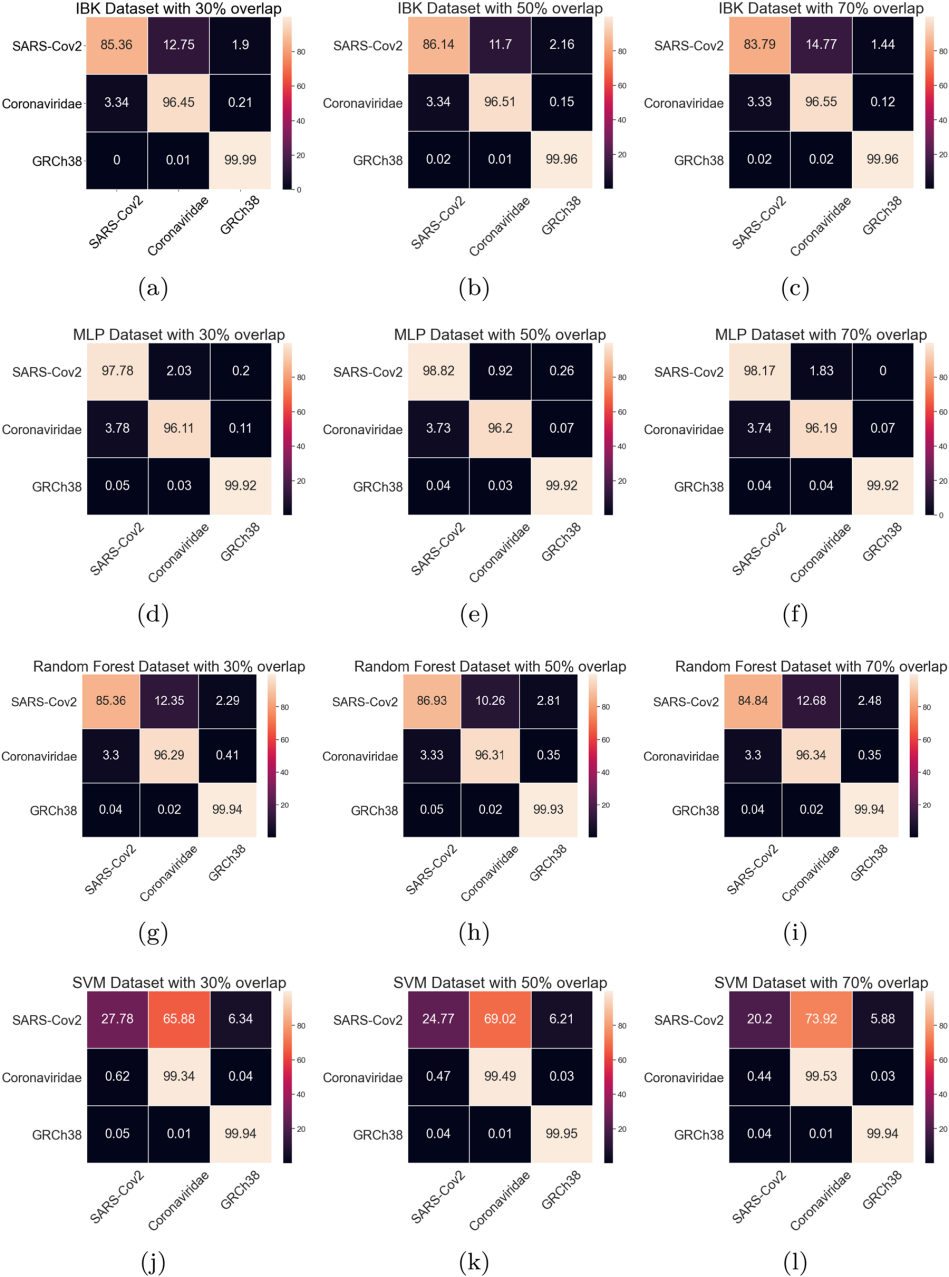
Average Confusion matrices for IBK, MLP, Random Forest and SMV in the SARS-CoV-2 test scenario.

Aiming to better evaluate the results of the classifiers in the SARS-CoV-2 test task, all the confusion matrices for IBK, MLP, Random Forest, and SVM classifiers are shown on Fig. 19. IBK and Random Forest classifiers presents a confusion between SARS-CoV-2 and Coronaviridae that varies from 10.26% (Fig. 19h) to 14.77% (Fig. 19c). This outcome is even worse for SVM classifier since most of the SARS-CoV-2 examples are misclassified as Coronaviridae. By confusion matrix analysis, the MLP classifier has lower confusion rates between SARS-CoV-2 and Coronaviridae. The results from MLP classifier in the dataset with 50% overlap (Fig. 19e) shows 99.92% average true positive rate for GRCh38 class, and 98.82% for the SARS-CoV-2. For the Coronaviridae class, this classifier achieves 96.2%, while only 3.73% of Coronaviridae examples are misclassified as SARS-CoV-2. Table 5 shows the sensitivity, specificity and ROC Area for each of the classes for this MLP classifier.

## Discussion

Considering the proposal for the representation of genomic sequences, the good classification results using classic machine learning methods show evidences that the pseudo-convolutional method for feature extraction proposed in this work may be sufficient to guarantee the identification of viruses from the transcribed DNA with high sensitivity, specificity and area of the ROC curve. The Random Forest classifier obtained the best overall performance for multiclass scenarios, while the MLP classifier presented the best results for scenarios with fewer classes.

Evaluating the parameters for the proposed sequence-based feature extraction method, dividing the virus genome sequence into four folders ($n = 4$) seems to be sufficient to produce representative characteristics. Regarding the percentage of overlaping, the proposed feature extraction method is not very sensitive to this parameter, although 30% to 50% seems to be sufficient to produce good characteristic representations.

The first multiclass scenario (with 25 virus classes) is a scenario designed to demonstrate extreme difficulty. It is highly unlikely that many of the virus families present in the classification are diagnostic possibilities, since the forms of contagion can be different and the diseases can manifest themselves through very different symptoms. However, the Random Forest classifier achieved sensitivity and specificity above 0.9 for many classes. For classes with lower sensitivity, the confusion matrix shows that most confusions are particular between two families of viruses. For example, Filoriviridae is the class with the lowest sensitivity rate (0.23). However, checking the confusion matrix, on average 76.27% of Filoriviridae are mistakenly classified as Ebola virus. There is no other significant confusion for Filoriviridae. Then it is possible to design a cascade of classifiers to solve this specific confusion between two viruses.

A particular class of virus is the Pharma Viridae. This family has only 42 samples in the data set (30 used for training and 12 for testing). Even with this small number of samples in the multiclass scenario, the significant incorrect classifications for Pharma Viridae are Henteraviridae (22.78%) and Peribunyavirida (35.26%). With a larger sample size for Pharma Viridae, classifiers could find a better decision frontier at this false-negative rate. However, for this particular class, cascading classifiers of three classes can be evaluated to deal with these types of errors.

**Figure 20:**
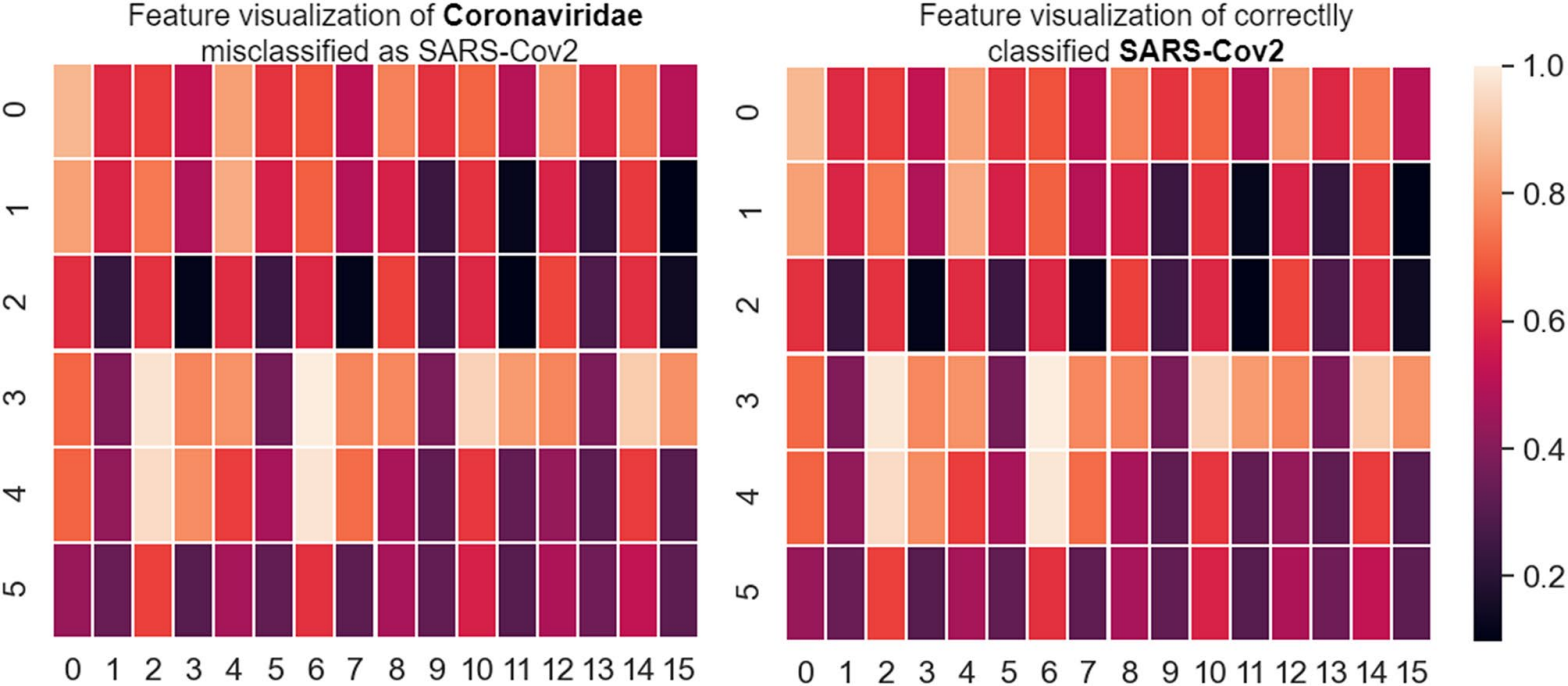
Feature visualization for selected SARS-CoV-2 and Coronaviridae sample.

Regardless of the characteristics extraction parameters or even the classifier used, there are still 3–4% of Coronaviridae samples classified incorrectly as SARS-CoV-2. However, this is an expected result, since SARS-CoV-2 belongs to the family Coronaviridae. We artificially separate the SARS-CoV-2 sequences from the Coronaviridae family, in order to verify whether the proposed pseudo-convolutional representation of genomic sequences would be able to help detect differences between SARS-CoV-2 and the other human coronaviruses . Thus, visualizing the extracted features, we found some samples of SARS-CoV-2 and Coronaviridae that cannot be distinguished, as shown in Fig. 20. Therefore, it is difficult for any classifier to ideally separate these two classes.

In this work we presented a novel method to represent DNA sequences by using pseudo-convolutions and co-occurrence matrices. With this method, we were able to represent hundreds of thousands of DNA sequences from 24 virus families. Then we separated SARS-CoV-2 sequences from the Coronaviridae family and demonstrated that our model is able to differentiate all virus families present on our database. SARS-CoV-2 was discriminated from virus families other than Coronaviridade and even from other coronaviruses with very high sensitivity and specificity.

Our goal was to show the ability to optimize the molecular diagnosis of Covid-19 by combining RT-PCR, Covid-19’s gold standard diagnostic method, and our pseudo-convolutional method to identify DNA sequences from SARS-CoV-2 from others candidate viruses without high computational cost bioinformatics methods, such as multiple sequence alignment.

From the results obtained, we can assume that the proposed method is able to characterize DNA sequences from SARS-CoV-2 transcribed from the RT-PCR process. This new representation of DNA sequences can be used successfully as a feature extraction stage for fully connected networks, in order to use the deep learning philosophy or other classical classification architectures. The assessment of the proposed approach in real test scenarios, necessarily reduced to a limited set of candidate virus families and DNA from healthy human samples, also showed high sensitivity (greater than 0.988) and specificity (greater than 0.998). Consequently, other researchers can use our solution and our methods to improve their results and diagnose Covid-19 and other viral diseases considering a considerably high number of candidate viruses with very high accuracy, precision, sensitivity and specificity.

The virus identification system proposed in this work was designed for clinical application, in clinical analysis laboratories. Potential users are biomedical technicians and other health professionals responsible for analyzing DNA samples from RT-PCR tests. The system was proposed as an alternative to web systems based on DNA sequence alignment, which can be used in a similar way from the point of view of potential users, i.e. biomedical technicians. The main limitation of the system is its restriction for analyzing DNA sequences of viruses, since many viruses are expressed as RNA, and not as DNA. The system is in the process of being implemented at the Biomedical Computing Laboratory of the Federal University of Pernambuco. As future work, we propose to adapt the proposed pseudo-convolutional network approach to RNA sequences, providing potential users to identify viruses both by their expression in RNA or in DNA and by the translation of RNA into DNA, in the case of RNA viruses.

## Methods

### Classifiers

To verify the efficiency of the proposed pseudo-convolutional method in extracting characteristics from the genome, classical and well-established classifiers were chosen in the state of the art of machine learning. In the same way that in deep architectures of artificial neural networks the final classifier is relatively simple, we chose not to introduce new classifier architectures, to keep the focus on the proposal of representing DNA sequences. The following classifiers were chosen:

#### Random Forest

**Figure 21:**
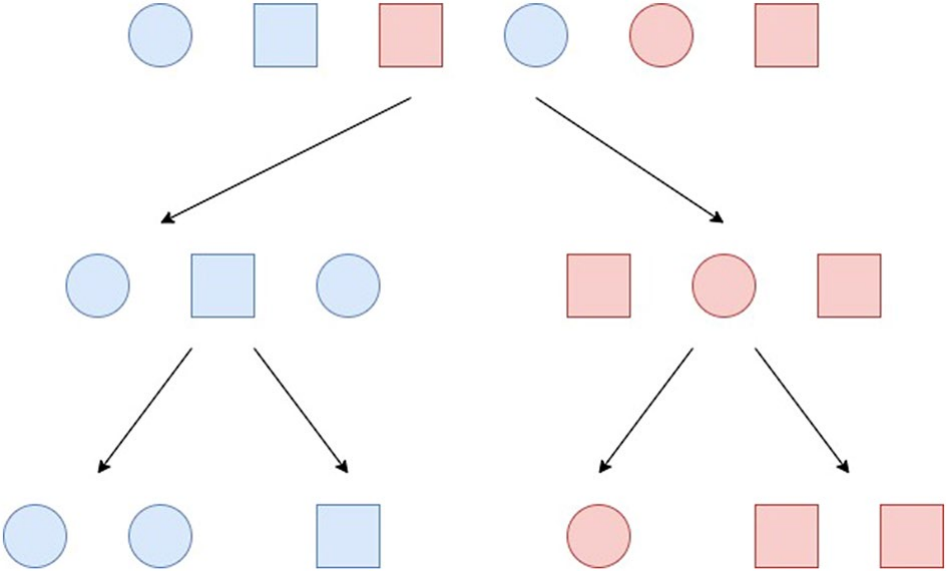
This decision tree example illustrates the classification of samples by two different features, colour and then shape.

Random Forests are based on decision tree committees organized in bagging^47^. Decision trees, as illustrated in Fig. 21, separate data iteratively, testing one property at a time. The resulting sheets represent the most specific category. The root represents the raw data. The random forest is built with many of these trees, all with their own class prediction for any input provided. The most voted class is the departure of Random Forest. Random Forests have been used to solve a plethora of biomedical problems, specially to develop intelligent systems to support diagnosis^48–50^.

As the most relevant characteristics to determine the decision boundary between classes of virus DNA sequences are unknown, Random Forests can be powerful methods for classification, as they are able to verify many relevant properties through their different trees. In the bagging process, each tree receives a version of the training set with a reduced number of attributes. Thus, it is possible to build decision criteria that take into account only a few attributes and these criteria can be winners in the vote, determining the final decision of the classifier.

#### Naive Bayes classifier

This machine learning model is based on Bayesian Decision Theory^51^. Given a set of classes $\Omega = \{\omega _1, \omega _2, \dots , \omega _m\}$ and a universe $\mathbb {R}^n$ of feature vectors $\mathbf {x} = (x_1, x_2, \dots , x_n)^T$ that model instances of a representation universe, the probability of deciding for class $w_i$ given an attribute vector $\mathbf {x}$ is given by:

2 $$\begin{aligned} P(\omega _i|\mathbf {x}) = \frac{p(\mathbf {x}|\omega _i)P(\omega _i)}{p(\mathbf {x})}, \end{aligned}$$

where

3 $$\begin{aligned} p(\mathbf {x}) = \sum _{j=1}^m p(\mathbf {x}|\omega _j)P(\omega _j), \end{aligned}$$

whilst $P(\omega _i)$ is the a priori probability of $\omega _i$ and $p(\mathbf {x}|\omega _i)$ is the density probability distribution of $\mathbf {x}$ given the class $\omega _i$. The decision is given according to the following rule, called the maximum probability hypothesis:

4 $$\begin{aligned} k = \arg \max _i \{P(\omega _i|\mathbf {x})\} \Rightarrow \mathbf {x}\in \omega _k. \end{aligned}$$

It is called naive because it assumes independence in the features that lead to the events, i.e. given a feature vector $\mathbf {x} = (x_1, x_2, \dots , x_n)^T$, the features $x_i$ and $x_j$ are statistically independent, since $i\ne j$. Then, we can have the following:

5 $$\begin{aligned} p(\mathbf {x}|\omega _i) = \prod _{j=1}^n p(x_j|\omega _i). \end{aligned}$$

Consequently, the discriminant functions to model each class, $P(\omega _i|\mathbf {x})$, are defined as following:

6 $$\begin{aligned} P(\omega _i|\mathbf {x}) = P(\omega _i) \frac{\prod _{j=1}^n p(x_j|\omega _i)}{\sum _{j=1}^m \left[ P(\omega _j) \prod _{k=1}^n p(x_k|\omega _j)\right] }, \end{aligned}$$

where $p(x_j|\omega _i)$ are usually modeled as Gaussian probability density functions:

7 $$\begin{aligned} p(x_j|\omega _i) = \frac{1}{\sigma _{j,i} \sqrt{2\pi }}e^{-\frac{1}{2}\left( \frac{x_{j,i} - \mu _{j,i}}{\sigma _{j,i}}\right) ^2}, \end{aligned}$$

whilst $$x_j|\omega _i \sim N(\mu _{j,i}, \sigma _{j,i}^2)$$, $$\forall i,j = 1, 2, \dots , n$$.

Naive Bayesian classifiers are commonly tested against other classifiers in diagnosis support solutions^48–50^. Furthermore, they assume all features/predictors have an equal weight. This approach could be beneficial because it explores the possibility that the genomes have characteristics that are not correlated. Should that be the case, this classifier might yield good results.

#### Instance-based learner

**Figure 22:**
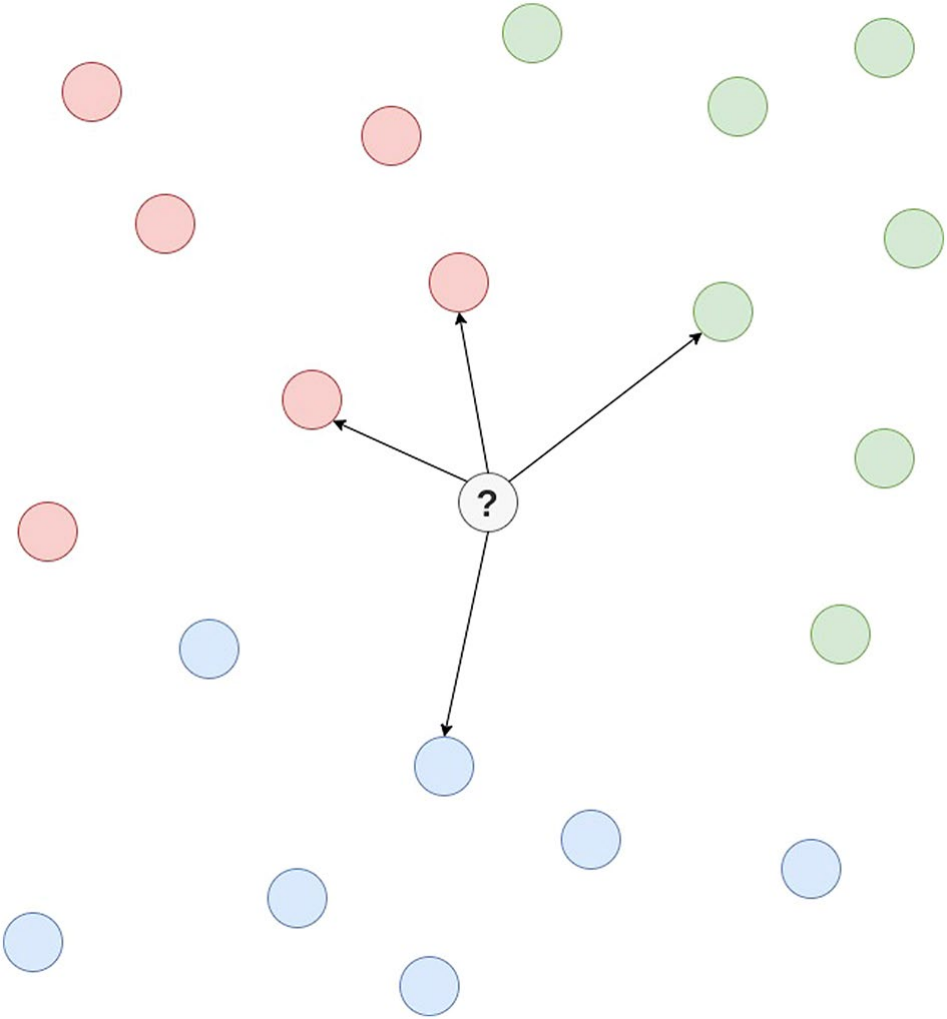
Simplified scheme to illustrate the training process as defined by the instance-based learner.

In this algorithm, also known as IBK^52^, a model is not built. It stores the training set. Given a sample, the prediction is made by calculating the distance *k* between that instance and the training set instances, as shown in Fig. 22. The distance is determined by some similarity criteria. Thus, this algorithm makes the decision based on the search for instances similar to the input instance. Thus, it may be able to identify genome sequences belonging to the same virus.

#### Multilayer perceptrons

**Figure 23:**
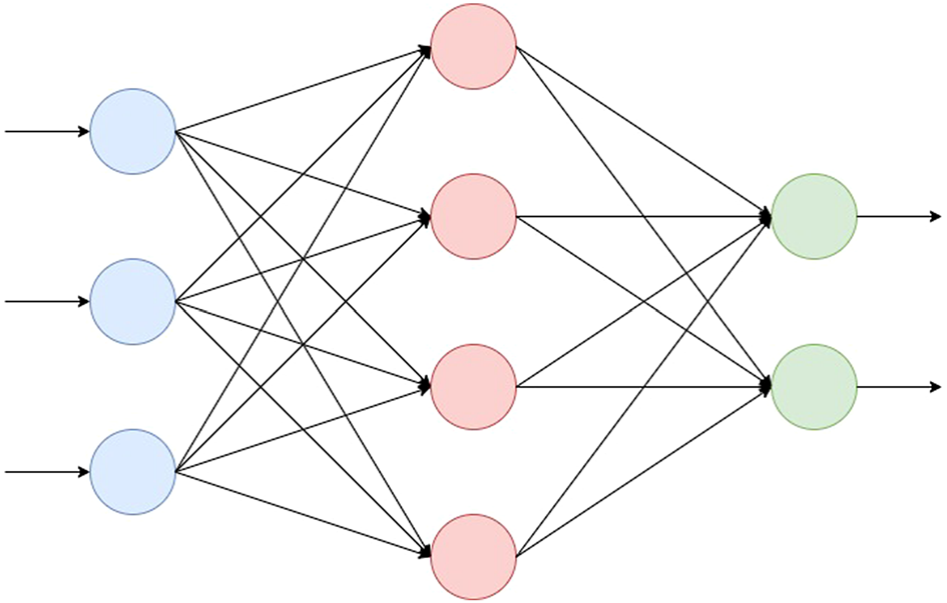
A multilayer perceptron with three layers of neurons.

This classifier, shown in Fig. 23, is an artificial neural network designed to solve nonlinearly separable problems^53^. Each artificial neuron has weights that multiply the input, which in turn is processed by an activation function to generate the output. The weights are adjusted until the net can satisfy a certain precision at the output. In this way, he could identify the characteristics that are particular to each class. Multilayer perceptrons have been used to several biomedical problems, specially to develop intelligent systems to support diagnosis^48–50,54^.

#### Support Vector Machines

**Figure 24:**
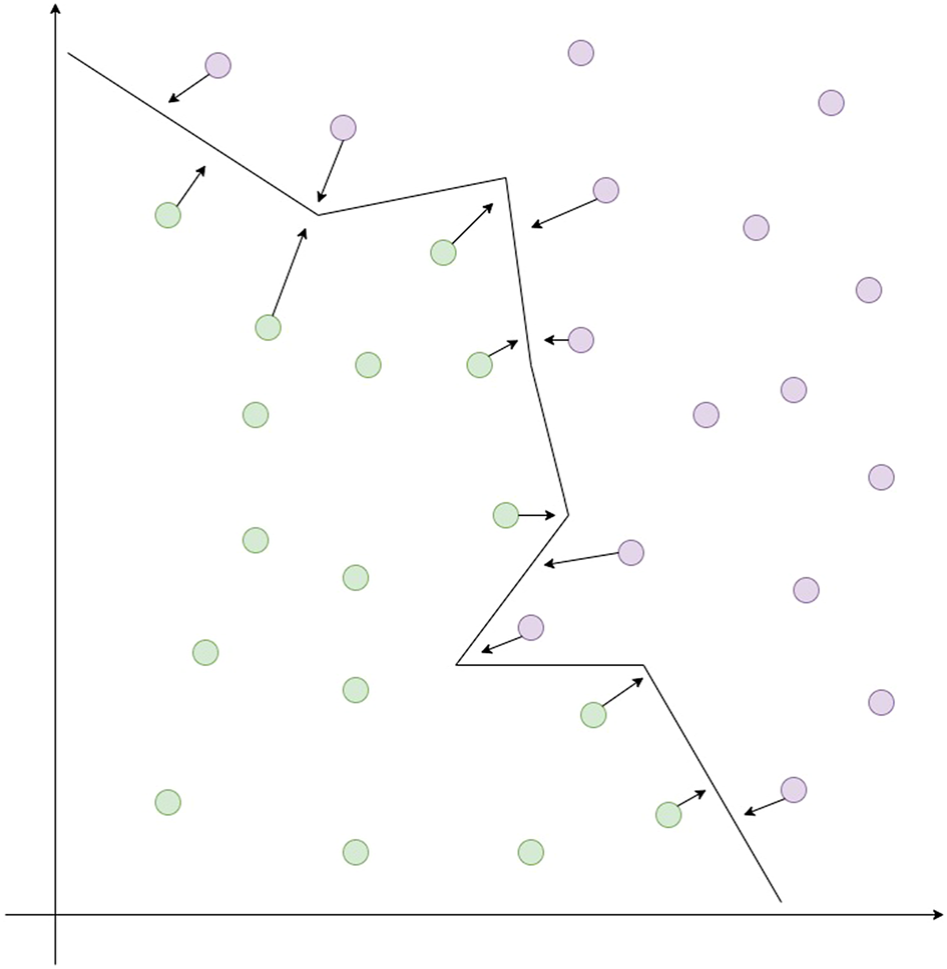
A binary classification problem, wherein the hyperplane created by the support vector machine has 2 dimensions.

Support Vector Machines (SVMs) were originally created for classification tasks where classes can be separated linearly, that is, by means of hyperplanes^55^. Through search algorithms, such as the Sequential Minimum Optimization Algorithm (SMO), vectors of characteristics of each class of the training set are chosen as supports. These support vectors are used to find the optimal hyperplan of separation of classes, according to the scheme of Fig. 24. To deal with nonlinear problems, SVMs use kernel transformations, which take the original attribute vectors to other representation spaces. According to the Coverage Theorem, the nonlinear mapping of attribute vectors in representation universes with more dimensions makes it more likely to find linear solutions to discriminate classes. SVMs have been applied to solve several biomedical problems, specially on developing intelligent systems for diagnosis support^48–50,54^. Since SVMs are commonly used in biomedical problems, presenting good results, it is reasonable to evaluate a considerable amount of SVM configurations and kernels to solve our problem considering the sequence representation method presented in this work.

### Database

Twenty-four different virus families were used to evaluate the efficiency of the feature extraction method, including the SARS-CoV-2 famility: Coronaviridae^56^. However, to separate SARS-CoV-2 from other coronaviruses, we used SARS-CoV-2 sequences as a separate class. Data was obtained from the NIAID Virus Pathogen Database and Analysis Resource (ViPR)^46^, which features multiple whole-genome sequences (WGS) from several viruses. Table 6 displays the number of examples per virus famliy for each of the selected viruses. Human participants were not involved in our research. No demographic data were collected as well.

**Table 6:** Number of instances in each class of virus.

Virus	Instances
Phasniaviridae	42
SARS-CoV-2	171
Hepeviridae	643
Poxviridae	697
Ebola virus	768
Nairoviridae	7977
Filoviridae	869
Zika virus	919
Lassa virus	1110
Peneunioviridae	1831
Arenaviridae	1840
Togaviridae	1983
Caliciviridae	2010
Paramyxoviridae	2609
PJiabdoviridae	2621
Hantaviridae	2785
Phenuiviridae	3089
Peribuiryaviridae	3245
Coronaviridae	3256
Enterovirus	3784
Dengue	5885
Picomaviridae	5894
Flaviridae	14,658
Reoviridae	62,454
Hepatitis C virus	216,223

The viruses have different sample sizes, ranging from 42 sequences, as for Phasmaviridae, to 216,223 nucleotide sequences, for Hepatitis C. The bar graphs below depict the distribution of sample sizes in both a linear and a logarithmic scale.

The second dataset used in this paper is from the Genome Reference Consortium^57^. Its purpose was to represent the human genome. We used 103,959 sequences.

### Feature selection

To evaluate the relevance of the features generated by the DNA sequences representation method proposed in this work, we used meta-heuristic optimization methods. Populations of 20 individuals were used, evolving in 20 generations. As an objective function, we use a decision tree, trained and tested using 10-fold cross validation. Each individual represents the attributes used in the classification by means of a binary vector, where 1 models the presence of that attribute, while 0 represents its opposite. We employed meta-heuristic libraries developed in Java for Weka data mining platform^58^. We adopted the following feature selection methods:

- Ant Colony Search, with chaotic coefficient of 4.0, chaotic type of logistic map, evaporation of 0.9, heuristic of 0.7, bit-flip mutation, mutation probability of 0.01^59–61^;
- Bat Search, with chaotic coefficient of 4.0, chaotic type of logistic map, frequency of 0.5, loudness of 0.5, bit-flip mutation, mutation probability of 0.01^62–64^;
- Bee Colony Search, with chaotic coefficient of 4.0, chaotic type of logistic map, objective by merits, radius damp of 0.98, radius mutation of 0.8, report frequency of 20, bit-flip mutation, mutation probability of 0.01^65–67^;
- Cuckoo Search, with chaotic coefficient of 4.0, chaotic type of logistic map, objective by merits, pa rate of 0.25, sigma of 0.69657, report frequency of 20, bit-flip mutation, mutation probability of 0.01^68–70^;
- Elephant Search, with chaotic coefficient of 4.0, chaotic type of logistic map, objective by merits, report frequency of 20, bit-flip mutation, mutation probability of 0.01^71–73^;
- Evolutionary Search, with crossover probability of 0.6, mutation probability of 0.1, bit-flip mutation, random initialization, generational replacement operator, report frequency of 20, survivor selection by tournament^74–76^;
- Firefly Search, with chaotic coefficient of 4.0, chaotic type of logistic map, objective by merits, report frequency of 20, bit-flip mutation, mutation probability of 0.01, absorption of 0.001, beta minimum of 0.33^77–80^;
- Rhinoceros Search, with chaotic coefficient of 4.0, chaotic type of logistic map, objective by merits, report frequency of 20, bit-flip mutation, mutation probability of 0.01^81,82^;
- Wolf Search, with chaotic coefficient of 4.0, chaotic type of logistic map, objective by merits, report frequency of 20, bit-flip mutation, mutation probability of 0.01, absorption of 0.001, beta minimum of 0.33, escape of 0.8^83–85^;
- Particle Swarm Optimization, individual weight of 0.34, inertia weight of 0.33, mutation probability of 0.01, report frequency of 20, social weight of 0.33^86–89^.

All feature selection parameters were defined empirically, taking into account the most commonly used parameters values.

Each attribute selection method returns the probability of relevance for each of the 97 attributes extracted from the base of virus DNA sequences. At the end, we checked the agreement between the methods, seeking to find a consensus among the attributes selected by each method. Interestingly, all methods returned 100% relevance for each of the 97 attributes, showing that the attributes generated by the DNA sequence representation method proposed in this work generate equally relevant and distinctive attributes, with no representation redundancies.

### Experiment setups

In order to validate our proposal, we designed four experiments based on theoretical and practical situations in which it is necessary to identify SARS-CoV-2 among a determined set of virus candidates represented by transcripted DNA sequences: (a) multiclass classification; (b) binary classification; (c) virus with similar symptoms; and (d) real test scenario. These experiments are described as following.

#### Multiclass classification

The purpose of this experiment is to identify the SARS-CoV-2 virus considering 24 virus families, including its own family: Coronaviridae. The SARS-CoV-2 sequences were separated from the Coronaviridae family to differentiate SARS-CoV-2 from other human coronaviruses. The complete list of virus families is shown in Table 6. All 25 classes in Table 6 were used to build the database, which was divided into a training set and a test set. For classes with more than 500 instances, the training set consisted of 500 instances, while the remaining instances were allocated to the test set. Classes with less than 500 distances were divided into 70% of instances for training and 30% for testing. In addition, the *n* attribute extraction hyperparameter was set to 4. The sequence overlap was tested for 30%, 50% and 70%. The general objective of this experiment is to evaluate the ability of the proposed sequence representation algorithm to classify virus DNA sequences transcribed from RT-PCR in the worst case scenario, where there is no knowledge about the virus present in the sample. This is a purposefully difficult scenario, although unlikely from a practical point of view, but one that tends to test the performance of the proposal in the worst case scenario.

#### Binary classification

This test was used to analyze the efficiency of the proposed method in differentiating SARS-CoV-2 from other viruses belonging to the Coronaviridae family. Viruses in the same family can be potentially challenging to classify when compared, given that the structural similarity is considerably high. In this scenario, the two with their genomes are contrasted only with each other. The training and testing division was carried out exactly as in the multiclass evaluation. The *n* feature extraction hyperparameter was set to 4 and the overlap was set to 30%, a percentage considered satisfactory in the multiclass experiment.

#### Viruses with similar symptoms

A third test was planned to classify viruses with symptoms similar to SARS-CoV-2. This test scenario is useful for situations where it is necessary to determine whether a patient has been infected with SARS-CoV-2 but has symptoms that may indicate infection with other viruses, which have similar symptoms. Four classes were established: SARS-CoV-2; Coronaviridae; Paramyxoviridae; Peneumoviridae, Hantaviridae, Enterovirus, and Nairoviridae. The criteria for building the training and test sets and for choosing the *n* hyperparameter were maintained as in the previous tests. The overlap was defined as 30%, 50% and 70%.

#### Real test scenario

This test was designed to reproduce the most common scenario of using RT-PCR to diagnose Covid-19. This scenario included three classes: the human genome, obtained from the Genome Reference Consortium^57^, SARS-CoV-2 and the other viruses in Table 6. It tests the actual use case of the proposed method, in which SARS-CoV-2 must be identified between the human genome and other viruses. The training and test sets were built in the same way as in the previous scenarios. The *n* hyperparameters has been maintained. The overlap was tested for 30%, 50% and 70%.

### Metrics

The following metrics were used to evaluate the classification performance of our proposal in all test scenarios:

- Confusion Matrix
  The confusion matrix provides a straightforward framework for portraying the model output. The lines represent the prior knowledge and the columns represent the results predicted by the model. *n* expresses the total number of instances. Each line, when added, is equivalent to the total number of instances per class. The number of correctly classified instances can be obtained by adding all the elements of the main diagonal. On the other hand, the number of instances classified incorrectly is obtained by adding the elements outside the main diagonal.
- Accuracy
  The accuracy describes the rate of correct classification of instances and is the most commonly used metric in machine learning. Considering a confusion matrix $T=[t_{i,j}]_{n\times n}$ for a classification task with *n* classes, in which *i* denotes the index of the *i*th true class and *j* points to the index of the class associated to the classification decision, the *j*th class, the accuracy is defined as following:
  8 $$\begin{aligned} \mathrm {Accuracy}=\rho _v=\frac{\sum _{i=1}^n t_{i,i}}{\sum _{i=1}^n \sum _{j=1}^n t_{i,j}}. \end{aligned}$$
- Kappa Coefficient
  The Kappa Coefficient ($\kappa$) assesses the relation between the classified instances. It is defined as:
  9 $$\begin{aligned} \kappa =\frac{\rho _v-\rho _z}{1-\rho _z}, \end{aligned}$$
  where
  10 $$\begin{aligned} \rho _z=\frac{\sum _{i=1}^{m}(\sum _{j=1}^{m}t_{i,j})(\sum _{j=1}^{m}t_{j,i})}{(\sum _{i=1}^{m}\sum _{j=1}^{m}t_{i,j})^2}. \end{aligned}$$
- Precision
  Precision indicates the proportion of positive and correct classification, and is thus calculated:
  11 $$\begin{aligned} \mathrm {Precision}=\frac{TP}{TP+FP}, \end{aligned}$$
  where TP is the number of true positives and FP is the amount of false positives.
- Recall
  Recall measures the proportion of actual positives correctly classified by the model. It is computed by:
  12 $$\begin{aligned} \mathrm {Recall}=\frac{TP}{TP+FN}, \end{aligned}$$
  where FN is the number of false negatives.
- Sensitivity
  The sensitivity, or True Positive Rate, is given by:
  13 $$\begin{aligned} TPR=\frac{TP}{TP+FN}. \end{aligned}$$
- Specificity
  The specificity, or True Negative Rate (TNR), if defined as following:
  14 $$\begin{aligned} TNR = \frac{TN}{TN+FP}, \end{aligned}$$
  where TN is the number of true negatives.
- Area Under the ROC Curve
  The Receiver Operating Characteristic (ROC) curve is a graph that plots the True Positive Rate (TPR) and False Positive Rate (FPR) of classification for different thresholds. The FPR is defined by:
  15 $$\begin{aligned} FPR=\frac{FP}{FP+TN}. \end{aligned}$$
  Thus, the Area Under the ROC Curve (AUC) measures performance for all possible thresholds of classification in a given model, and therefore it portrays the quality of results independently of it.

### Human or animal subjects

This article does not contain any studies with human or animal subjects.

## Data Availability

The main data supporting the results in this study are available within the paper. Data was obtained from the NIAID Virus Pathogen Database and Analysis Resource (ViPR)^46^.

## Code availability

The custom code is currently available on request.
